# Mitochondrial Genome Insights into Evolution and Gene Regulation in *Phragmites australis*

**DOI:** 10.3390/ijms26020546

**Published:** 2025-01-10

**Authors:** Jipeng Cui, Qianhui Yang, Jiyue Zhang, Chuanli Ju, Suxia Cui

**Affiliations:** 1 College of Life Sciences, Capital Normal University, Haidian District, Beijing 100048, China; cuijipeng0220@163.com (J.C.); y2776834662@126.com (Q.Y.); zhang_jiyue9858@163.com (J.Z.); chlju@cnu.edu.cn (C.J.); 2 Beijing Key Laboratory of Plant Gene Resources and Biotechnology for Carbon Reduction and Environmental Improvement, Beijing 100048, China

**Keywords:** *Phragmites australis*, mitogenome, sdh4, mtDEG

## Abstract

As a globally distributed perennial Gramineae, *Phragmites australis* can adapt to harsh ecological environments and has significant economic and environmental values. Here, we performed a complete assembly and annotation of the mitogenome of *P. australis* using genomic data from the PacBio and BGI platforms. The *P. australis* mitogenome is a multibranched structure of 501,134 bp, divided into two circular chromosomes of 325,493 bp and 175,641 bp, respectively. A sequence-simplified succinate dehydrogenase 4 gene was identified in this mitogenome, which is often translocated to the nuclear genome in the mitogenomes of gramineous species. We also identified tissue-specific mitochondrial differentially expressed genes using RNAseq data, providing new insights into understanding energy allocation and gene regulatory strategies in the long-term adaptive evolution of *P. australis* mitochondria. In addition, we studied the mitogenome features of *P. australis* in more detail, including repetitive sequences, gene Ka/Ks analyses, codon preferences, intracellular gene transfer, RNA editing, and multispecies phylogenetic analyses. Our results provide an essential molecular resource for understanding the genetic characterisation of the mitogenome of *P. australis* and provide a research basis for population genetics and species evolution in Arundiaceae.

## 1. Introduction

The current global warming has accelerated the degradation of wetlands and the extent of soil salinisation, exerting considerable pressure on wetland ecosystems and plants [1]. The *P. australis* is a globally distributed perennial Graminaceous plant with a broad ecological range. The phenotypic plasticity and genetic diversity of *P. australis* facilitate its adaptation to a range of challenging ecosystems, including saline wetlands, arid sand dunes, and upland meadows [2]. The extensive rhizome system of *P. australis* endows it with a robust water purification and salt enrichment capacity, instrumental in saline wetland restoration and upland soil and water conservation [3,4]. Furthermore, as a traditional Chinese medicine, *P. australis* rhizome significantly treats bacterial, inflammatory, and viral infections. It has been clinically employed by Chinese medicine practitioners for millennia [5,6,7].

Mitochondria and chloroplasts are semi-autonomous organelles that contain genetic expression systems in plant cells. In conjunction with the nuclear genome, the mitogenome plays a crucial role in respiration, cellular metabolism, apoptosis, and cytoplasmic genetics [8,9,10]. Given that the mitogenome evolves at a rate that is less than one-sixth that of the nuclear genome, it is a commonly employed tool in studying species evolution and phylogeny [11]. In contrast to the highly conserved quadripartite circular structure of plastid genomes, the structural conformation of plant mitogenomes is complex and variable, encompassing a range of forms such as circular, linear, branched, multichromosomal, and so forth [9,12,13]. The structure and conformation of plant mitogenomes can now be observed and studied with greater ease and precision using transmission electron microscopy and scanning electron microscopy. For instance, the mitogenome structure of Vigna radiata cotyledon tissue was found to undergo a gradual conformational change during seed germination [12].

As a result of more in-depth studies of the mitogenome, increased mitochondrial gene functions or regulations have been elucidated in greater detail. Plant mitochondrial proteomics studies have demonstrated that the expression levels of specific plant mitochondrial proteins can be influenced by abiotic stresses, such as temperature and flooding, e.g., mitochondrial electron transport chain complex I~V, uncoupling protein, etc. [14,15,16]. Mitochondria, as a primary site of respiration, represent a significant source of reactive oxygen species (ROS) within cells. The overexpression of the mitochondrial oxidation resistance protein AtOXR2 in Arabidopsis thaliana has increased plant biomass and seed yield while enhancing tolerance to ROS bursts from methyl viologen and high light stress [17]. Furthermore, mitochondria have been shown to play an essential role in plant immunity, with the ability to mediate the production of mitochondrial reactive oxygen species (mROS), reactive nitrogen species (RNS), and hormone signalling pathways in response to pathogen attack [18,19]. These findings suggest that mitochondrial genes are crucial and indispensable in the plant stress response.

The development of third-generation sequencing (TGS) technology, represented by PacBio High Fidelity sequencing and Oxford Nanopore sequencing technology, has significantly advanced plant genomics research and increased the data resources available for organelle genome assembly [20,21,22]. These TGS reads cover more complex recombination regions of the mitogenome and provide data to resolve the structural dynamics of the mitogenome and different isoforms. In this study, we filtered the organelle genome sequencing data among the genome sequencing data from the PacBio and BGI platforms. We assembled and characterised a complete structural profile of the mitogenome of *P. australis*. Based on transcriptome data, the validation of identified RNA editing sites was accompanied by identifying tissue-specific mitochondrial differentially expressed genes. Furthermore, molecular characteristics, including codon preference, repetitive sequences, intracellular gene transfer, and gene selection pressure within the mitogenome of *P. australis*, were meticulously delineated. Phylogenetic analyses based on several gramineous species’ mitogenomes and chloroplast genomes have demonstrated a close evolutionary relationship between *P. australis* and the Chloridoideae. Our results contribute to the understanding of the structure and function of the mitogenome of *P. australis* and provide usable data resources for evolutionary and genetic studies of *P. australis*.

## 2. Results

### 2.1. Assembly and Annotation of P. australis Mitogenome

**Figure 1:**
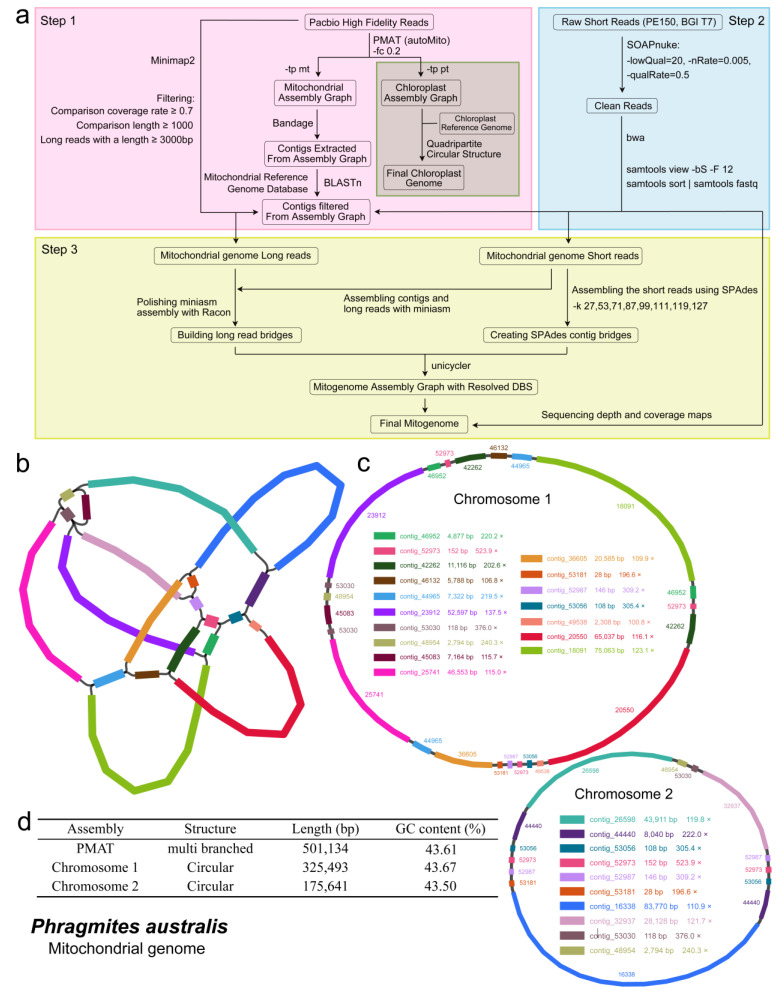
*P. australis* mitochondrial gene assembly process and structural characterisation. (**a**) Organelle genome assembly process from genome sequencing data. (**b**) *P. australis* mitogenome master graphs. (**c**) Two circle graphs were obtained by hybrid assembly using Unicycler. Each circle graph represents the ring molecules of one chromosome of the mitogenome. Fragment colours indicate the same contig sequence fragments as in the master graphs. (**d**) Basic mitogenome information.

A pipeline for filtering and assembling organelle genomes from whole genome sequencing data was designed through publicly available software or programs (Figure 1a). These mitogenome master graphs (MGs) using HiFi long-read sequencing data encompass the most complex repetitive sequences. Consequently, this organelle genome assembly is more accurate and complete and contains more potential genome conformations. In this work, mitogenome MGs (comprising 21 contigs) (Figure 1b) and chloroplast genome MGs (comprising 3 contigs) (Figure S1) with multiple branching structures were obtained. The mitochondrial sequencing data obtained after filtering were subjected to hybrid assembly (using both long and short reads data) using Unicycler software, which was employed to resolve the double-bifurcating structure (DBS) in the mitogenome MGs. The final assembly comprised two chromosomes of the *P. australis* mitogenome ring structure, designated as chromosome 1 and chromosome 2 (Figure 1c). The lengths of the two chromosomes were 325,493 bp and 175,641 bp, respectively, with GC contents of 43.67% and 43.50% (Figure 1d and Table S1). The accuracy of the mitogenome assembly was validated by mapping the long and short reads to the final assemblies (Figure S2). Furthermore, the chloroplast genome MGs were resolved into a quadripartite circular structure based on the *P. australis* reference chloroplast genome.

**Figure 2:**
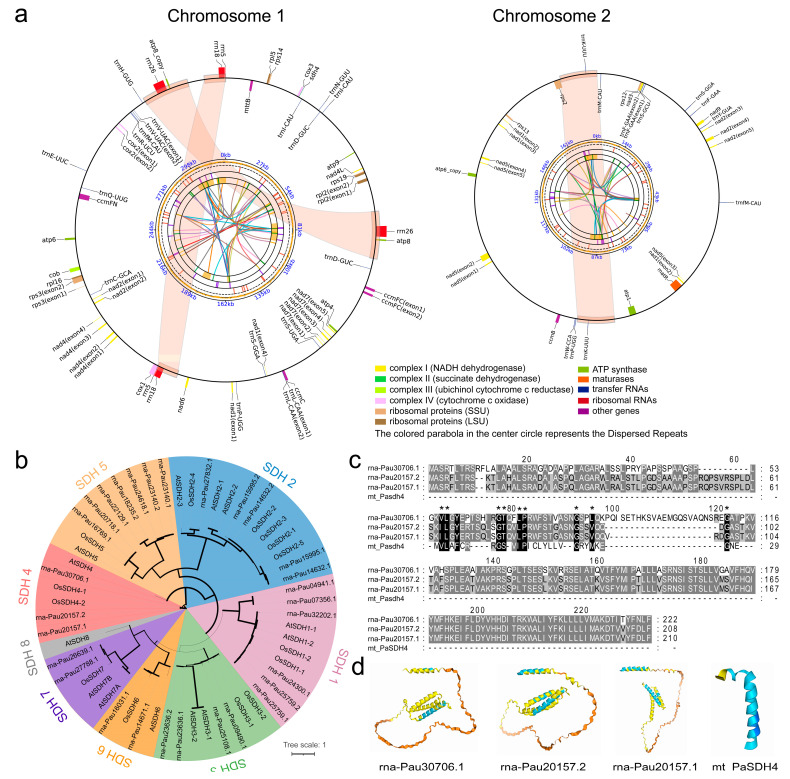
A schematic map of the mitogenome of *P. australis* and the identification of the succinate dehydrogenase subunit gene. (**a**) Schematic maps of the two circular chromosomes of the *P. australis* mitogenome, with the colour used to distinguish genes of different functional groups. The images illustrate the sequence of events from the inside out. (1) The relationship between dispersed repeat sequences. (2) The distribution of dispersed repeat sequences on the chromosome where yellow represents direct dispersed repeat sequences and green represents inverted dispersed repeat sequences. (3) The distribution of tandem repeat sequences on the chromosome. (4) The distribution of tandem repeat sequences. (5) The distribution of GC content on the chromosome. (6) The scale coordinate axis. (7) Genes located on the negative strand. (8) Genes located on the positive strand. (9) Orange shadows represent forward dispersed repeats that exceed the selection threshold. (**b**) Phylogenetic tree of the SDH gene family in the nuclear genome of *P. australis.* (**c**) Sequence comparison of 3 nuclear genome Nu_SDH4 transcripts with Mt_SDH4 in the mitogenome. (**d**) Structure prediction of Mt_SDH4 protein in 3 nuclear genome Nu_SDH4 transcripts versus mitogenome.

The annotation of the *P. australis* mitogenome yielded a total of 69 genes, comprising 36 protein-coding genes (PCGs), 27 transfer RNA genes (tRNAs), and 6 ribosomal RNA genes (rRNAs) (Figure 2a, Table 1). These protein-coding sequences account for 6.48% (32,496 bp) of the entire genome (Table S2), including ATP synthase genes (*atp1*, *atp4*, *atp6 (×2)*, *atp8 (×2)*, *atp9*), NADH dehydrogenase genes (*nad1*, *nad2*, *nad3*, *nad4*, *nad4L*, *nad5*, *nad6*, *nad7*, *nad9*), cytochrome c biogenesis genes (cob), ubiquinol cytochrome c reductase genes (*ccmB*, *ccmC*, *ccmFC*, *ccmFN*), cytochrome c oxidase genes (*cox1*, *cox2*, *cox3*), maturases (*matR*), transport membrane protein genes (*mttB*), a large subunit of ribosome genes (*rpl5*, *rpl16*, *rpl2*), a small subunit of ribosome genes (*rps2*, *rps3*, *rps12*, *rps13*, *rps14*, *rps19*), and succinate dehydrogenase subunit 4 (*sdh4*) (Figure 2a). It was unexpected that an Sdh4 gene (*mt_PaSDH4*) was retained in this *P. australis* mitogenome and that the sequence of this sdh4 gene partially overlapped with that of the *cox3* gene. Statistical analysis of the retention of sdh genes in published plant mitochondrial genomes was conducted, which revealed that the mitochondrial genomes of Rosaceae, Fabaceae, and Asteraceae retained a greater number of sdh4 genes (Figure S3a). In contrast, one sdh4 gene was retained in the mitochondrial genomes of the graminaceous species *P. australis* and *Avena longiglumis*, respectively (Figure S3a). The gramineous sdh4 was clustered in one cluster in the phylogenetic tree (Figure S3b). To ascertain whether a coevolutionary relationship exists between sdh4 genes in the mitochondrial and nuclear genomes, 29 transcripts of the *Nu_PaSDH* family were identified in the nuclear genome of *P. australis* using SDH family sequences from *Arabidopsis thaliana* and *Oryza sativa*. These transcripts contained three Nu_PSDH4 transcripts (Figure 2b). In comparison to the SDH proteins identified in the nuclear genome, the mt_PSDH4 sequence underwent a process of simplification and compression yet still retained a segment of the α-helical structure containing a conserved motif consisting of 29 amino acids (V/I L—F—G S/T—I/L P—G—L/V/M—G) (Figure 2c,d). The Ka/Ks ratios between all Nu_PSDH4 in the nuclear genome and mt_PSDH4 in the mitogenome were less than 1 (Table S3), suggesting that these *PaSDH4* genes were subjected to negative selective pressures in evolution after they entered into the nuclear genome and the accumulation of non-synonymous mutations was limited. These mitochondrial genes transferred into the nuclear genome were subjected to intense negative selection pressure in evolution, ensuring that these genes maintained the integrity of their original core functions after functional expansion or modification. In this study, we identified tRNA genes that contained seven duplicated tRNA genes and three tRNA genes that possessed introns (*trnL-CAA*, *trnF-GAA*, *and trnV-UAC*, respectively). The secondary structures of all these tRNA genes were predicted to be typical cloverleaf structures (Figure S4).

### 2.2. Analysis of Repetitive Sequences in the P. australis Mitogenome

**Figure 3:**
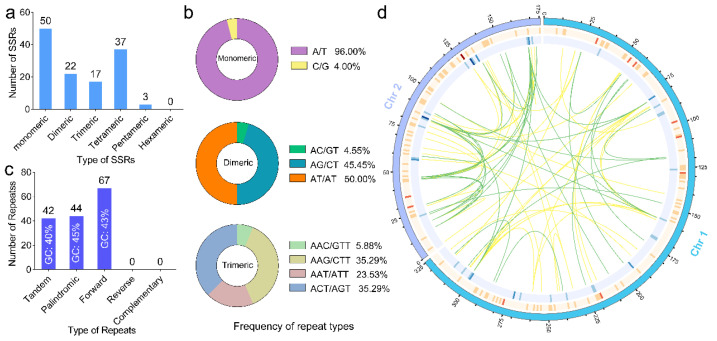
Repetitive sequences in the *P. australis* mitogenome. (**a**) Types and numbers of SSRs in the mitogenome of *P. australis*. The number of repeats in each category is shown at the top of the corresponding bar diagram. (**b**) The frequency of classified repeat types (considering sequence complementary) in monomeric, dimeric, and trimeric repeat types. (**c**) Statistics of repeat sequence types in the mitogenome of *P. australis*. (**d**) The figure of the distribution of repetitive sequences in the mitogenome of *P. australis*. The orange colour indicates the distribution density of SSRs, blue circles indicate the distribution density of tandem repeat sequences, inside lines indicate dispersed repeat sequences, yellow lines indicate forward repeats (F), and green lines indicate palindromic repeats (P).

Simple sequence repeat (SSR) molecular markers are a valuable tool for genetic diversity and population genetic variation analysis due to their high polymorphism and reliability. A total of 129 SSRs with a total length of 1525 bp were identified in the mitogenome of *P. australis*. However, no hexanucleotide repeat sequences were found (Table 2, Figure 3a). The most prevalent motifs for SSR loci in the mitogenome of *P. australis* were single and tetranucleotide repeats, which collectively accounted for 67.44% of the total. Single nucleotide repeats constituted the predominant motif type in *P. australis*, representing 38.76% of the total, followed by tetranucleotide repeats at 28.68%. It was found that, upon analysis of the SSR repeat motif frequencies (considering sequence complementarity), the A/T types accounted for 96% of the single nucleotide repeats. Furthermore, it was observed that motifs containing A or T in both dinucleotide repeats and trinucleotide repeats constituted the majority of repeat motif types, including AT/AT (50.00%) and AAG/CTT (35.29%) (Figure 3b). This finding is consistent with the high proportion of AT bases observed in the mitogenome of Gramineae (Table S1). These results suggest that the SSR repeats in the *P. australis* mitogenome have a distinct base preference (A or T) and may have influenced the overall base ratio of the mitogenome. Sequences of dispersed repeats in plant mitogenomes are essential for genomic structural variation and include forward repeats (F), reverse repeats (R), palindromic repeats (P), and complement repeats (C). As illustrated in Figure 3d, a total of 111 pairs of dispersed repeat sequences were identified in both chromosomes of the *P. australis* mitogenome. These dispersed repeat sequences contain 67 pairs of forward repeats and 44 pairs of palindromic repeats, with the longest repeats reaching 16,147 bp (forward repeats). The complementary repeats and reverse repeats were not detected in the mitogenome of *P. australis*. Furthermore, 42 instances of tandem repeats, ranging in length from 27 to 210 bp, were identified within the *P. australis* mitogenome (Figure 3c,d).

### 2.3. Ka/Ks Analysis

**Figure 4:**
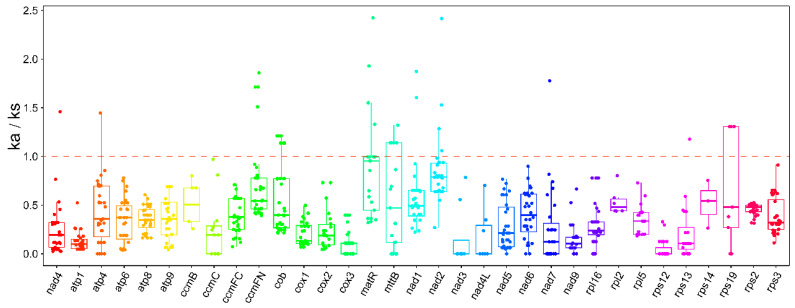
Boxplot of Ka/Ks ratios of *P. australis* with 32 other Graminaceous species.

Nucleotide mutations that do not result in an alteration of the amino acid sequence are referred to as synonymous mutations, whereas those that do are designated as non-synonymous mutations. Synonymous mutations are not subject to natural selection, whereas non-synonymous mutations are subject to natural selection. In order to evaluate the evolutionary exposure of PCGs in the mitogenome of *P. australis* to natural selection pressures, the ratio of the rate of non-synonymous mutations to the rate of synonymous mutations (Ka/Ks) was calculated for the mitogenomes of *P. australis* shared with 33 gramineous species (Figure 4). The Ka/Ks values of these PCGs were predominantly within the range of 0.2–0.7, with a mean value below 1. This suggests that the majority of PCGs in *P. australis* have been subjected to substantial purifying selection and exhibit a high degree of conservatism in comparison with gramineous species throughout the evolutionary process. Conversely, some gene pairs in *P. australis* exhibited Ka/Ks ratios greater than 1, including matR, mttB, nad2, and ccmFN, which may have been subject to positive selection during the evolutionary process.

### 2.4. Analysis of Codon Usage in PCGs

**Figure 5:**
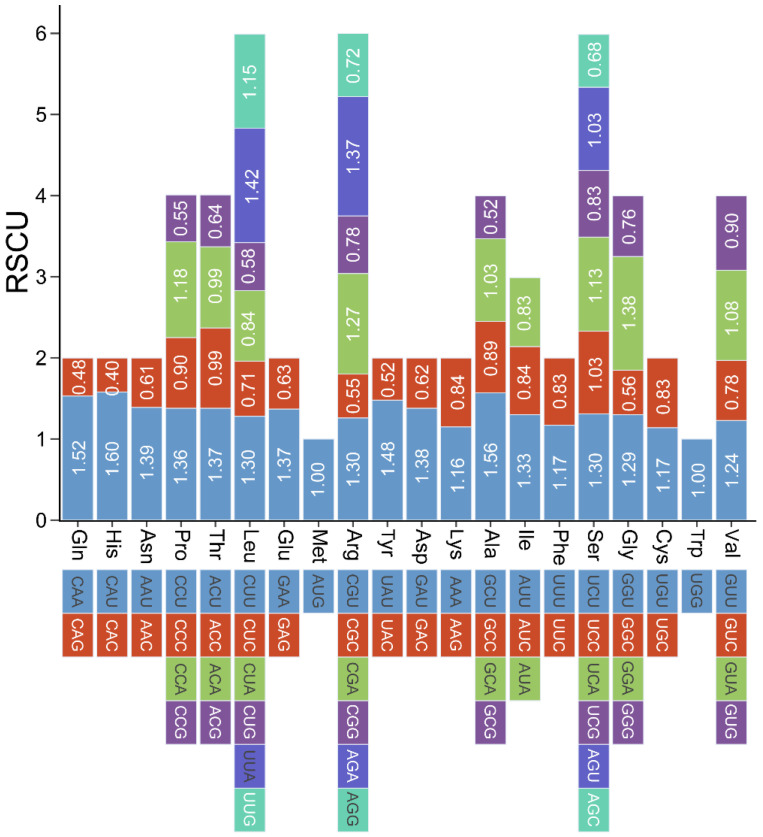
Analysis of the *P. australis* mitogenome relative synonymous codon usage. The coloured blocks below indicate the type of codon encoding each amino acid, and the coloured blocks above are the RSCU values for the corresponding codons.

To explore the unique codon usage preferences developed during adaptive evolution in *P. australis*, we performed codon usage analyses of PCGs in the reed mitogenome (Table S4). All PCGs used the start codon ATG as their start codon, with the exception of rpl16, which used GUG as its start codon. Three types of stop codons were identified, of which TAA was the most frequently used with 50.00%, followed by TGA (18.75%) and TAG (31.25%). The three most frequently used amino acids in *P. australis* mitochondrial PCGs were leucine (Leu) (10.73%), serine (Ser) (9.19%), and isoleucine (Ile) (7.61%). Cysteine (Cys) and tryptophan (Trp) were the least used, with 1.47% and 1.53%, respectively. The phenomenon of synonymous codon coding (degeneracy) exists in the transfer of an organism’s genetic information from mRNAs to proteins and usually occurs at the third base of the synonymous codon (wobbling) [23]. Relative synonymous codon usage (RSCU) was analysed by PCGs in the mitogenome of *P. australis* (Figure 5). The results showed that all synonymous codons with A or U at the third base of the codon had RSCUs greater than 1, except for methionine (Met) and tryptophan (Trp) (RSCU = 1), which showed a very significant codon preference. Among them, Gln, Glu, Lys, and Leu codons showed a preference for A at the third base, whereas Pro, Arg, Ala, Ser, Val, and Gly showed a significant preference for both A and T.

### 2.5. Intracellular Gene Transfer (IGT) of P. australis Mitogenome

**Figure 6:**
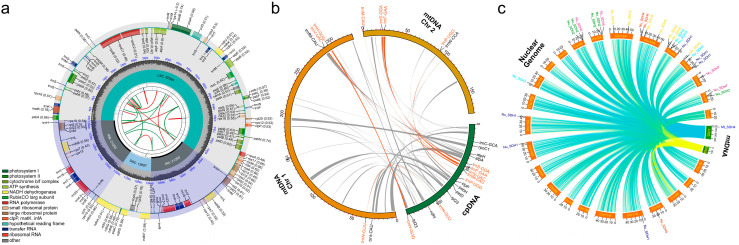
A schematic map of the MTPTs and NUMTs of the mitogenome of *P. australis*. (**a**) A schematic map of the chloroplast genome assembled in this study. (**b**) A Circos plot showing the MTPTs between the mitochondrial and chloroplast genomes. Genes labelled in the figure indicate genes contained in the MTPT, and the red text indicates genes in the MTPT with unchanged functions in the mitochondrial and chloroplast genomes. (**c**) A Circos plot showing NUMTs between 25 chromosomes of nuclear genomes and the mitogenome of *P. australis*. The arcs in B and C connect homologous sequence fragments between different genomes.

Intracellular gene transfer (IGT) denotes the phenomenon of transfer and fusion of genetic material between the genomes of a single cell. A total of 132 genes were annotated in the assembled chloroplast genome, comprising 85 protein-coding genes (PCGs), 8 ribosomal RNAs (rRNAs), and 39 transfer RNAs (tRNAs) (Figure 6a). In order to identify instances of intracellular gene transfer (IGT) between the mitochondrial and chloroplast genomes of *P. australis*, we employed the BLAST tool to identify mitochondrial plastid DNAs (MTPTs) based on the degree of similarity between mitochondrial and chloroplast sequences. As illustrated in Figure 6b, a total of 41 homologous fragments of the plastid genome were identified in the mitogenome, with lengths ranging from 36 bp to 4402 bp (28 in Chr1 and 13 in Chr2). The total length of these homologous fragments was 36,057, representing 7.20% of the mitogenome sequence. *P. australis* chloroplast genome sequence fragments were integrated into the mitogenome by inversion, recombination, and polymerisation to form MTPTs. Subsequently, a total of 27 complete genes were identified in these homologous fragments, including 17 genes in chloroplasts (9 PCGs genes: *rpoC1*, *atpA*, *atpH*, *clpP*, *psbH*, *petD*, *rpl23 (×2)*, *ndhl*; 8 tRNA genes: *trnC-GCA*, *trnS-GGA*, *trnF-GAA*, *trnV-UAC*, *trnM-CAU*, *trnP-UGG*, *trnN-GUU*, *trnH-GUG*) and 10 genes in mitochondria (*trnW-CCA*, *trnP-UGG*, *trnF-GAA*, *trnS-GGA*, *trnM-CAU*, *trnH-GUG*, *trnV-UAC*, *trnfM-CAU*, *trnN-GUU*, *trnI-CAU*) (Table S5). Here, we found that PCG genes in the plastid genome underwent loss of function or alteration after integration into the mitogenome, e.g., *rpoC1*, *atpA*, *atpH*, *clpP*, *psbH*, *petD*, *rpl23*, *ndhl,* etc. Furthermore, the sequences and functions of the seven transfer RNA (tRNA) molecules in the plastid genome, depicted in orange in Figure 6b, were preserved intact following their transfer and integration into the mitogenome. The migration direction and function of these MTPTs in the two organelle genomes will be the subject of further detailed and in-depth studies in the future. Nuclear mitochondrial DNA sequences (NUMTs) are preserved in the nuclear genome as evolutionary traces, providing valuable information for studying the evolutionary history of species [24]. As illustrated in Table S6 and Figure 6c, 4602 (278,534 bp) and 2246 (151,708 bp) homologous fragments transferred to the nuclear genome were identified in two *P. australis* mitogenome chromosomes, representing 85.85% of the mitogenome sequence, respectively. The NUMTs were distributed across 25 chromosomes of the *P. australis* nuclear genome, comprising a total of 6591 homologous fragments ranging in length from 43 bp to 16,534 bp, with a collective length of 1,123,956 bp. The abundance of these NUMTs suggests that gene transfer between the mitochondrial and nuclear genomes of *P. australis* is extensive and frequent.

### 2.6. Prediction and Validation of RNA Editing

**Figure 7:**
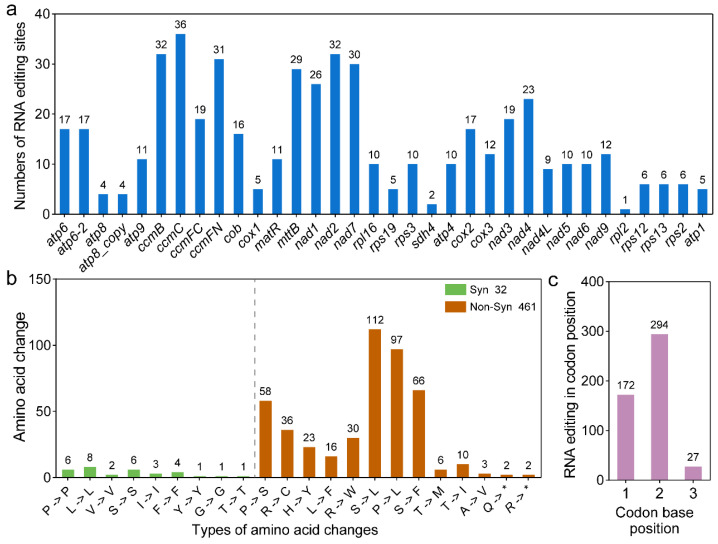
Characterisation of RNA editing sites in the mitogenome of *P. australis*. (**a**) Statistics of the number of predicted RNA editing sites in PCGs. (**b**) Amino acid changes caused by RNA editing in PCGs. * Indicates a codon that has been changed to a stop codon by RNA editing. (**c**) Location statistics of RNA editing in codon.

Deepred-Mt is a novel neural network capable of predicting the most major C to U editing sites in angiosperm mitochondria. Here, we used Deepred-Mt to identify RNA editing events in all PCGs with a probability cutoff greater than 0.9. A total of 493 RNA editing sites were identified in the *P. australis* genome, involving 34 PCGs (Table S7). Among these, the ccmC gene exhibited the highest frequency of RNA editing events, with 36 editing sites identified. In contrast, the rpl2 gene demonstrated a relatively low level of editing, with only a single event observed (Figure 7a). The number of amino acid changes resulting from RNA editing events was subsequently calculated. Of the identified RNA editing sites, 32 were synonymous (i.e., did not result in a change in the amino acid sequence) and 461 were non-synonymous (i.e., resulted in a change in the amino acid sequence). The most prevalent type of RNA editing in *P. australis* mitochondria was the conversion of serine (S) or proline (P) to leucine (L), representing 42.39% of all observed editing events (Figure 7b). It is noteworthy that the termination codons for atp6, atp9 and ccmFC were created by RNA editing in the *P. australis* mitogenome, with codon changes CAA → UAA, CGA → UGA, and CGA → UGA, respectively. Similarly, the start codons for nad1 and nad4L were created by RNA editing, which was achieved by editing ACG to AUG (Table S7). Furthermore, RNA editing events in *P. australis* PCGs predominantly occurred at codon position 2 (294, 59.63%) or position 1 (172, 34.89%), with 27 RNA editing sites identified exclusively at position 3 (Figure 7c). To ascertain the veracity of these RNA editing sites, we employed transcriptomic data from disparate tissues for validation purposes. Following the removal of SNP sites, the transcriptomic data identified a total of 584 RNA editing sites that were supported by the 168 RNA editing sites predicted by Deepred-Mt (Figure S5, Table S7) and, thus, can be considered as high-confidence RNA editing sites. Moreover, the RNA editing sites identified by these transcriptomic data exhibited notable tissue specificity, with 129, 106, and 137 tissue-specific RNA editing sites observed in leaves, aerial stems, and rhizomes, respectively. Of these, 14, 30, and 11 were classified as high-confidence RAN editing sites (Figure S5).

### 2.7. Analysis of Mitochondria-Related Differentially Expressed Genes (mtDEG)

**Figure 8:**
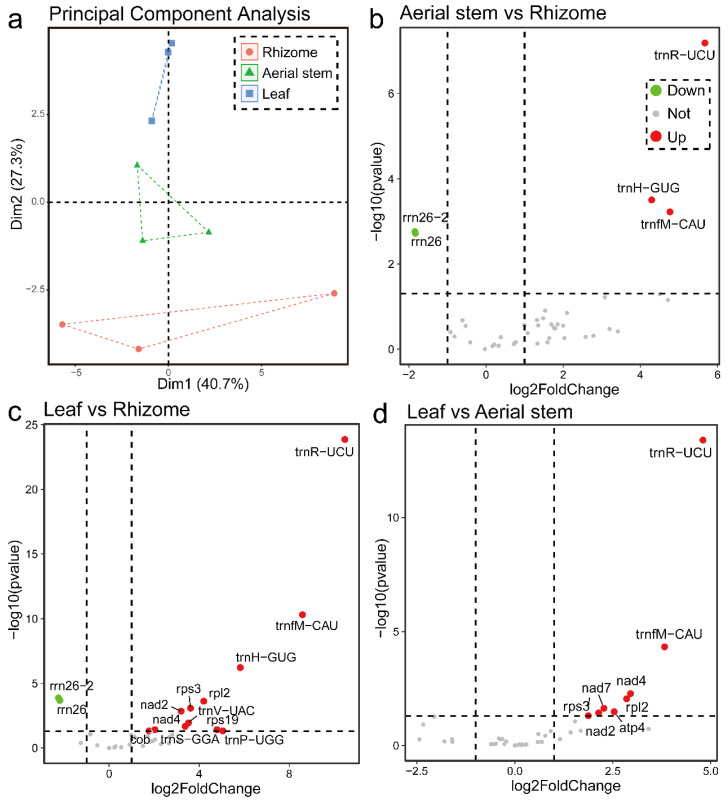
The mtDEG in the mitochondrial transcriptome of *P. australis*. (**a**) Principal component analysis of the transcriptome of *P. australis* leaf, aerial stem, and rhizome tissues. (**b**–**d**) Volcano plots showing mtDEG between different tissues, respectively, |log2FoldChange| > 1, *p* value < 0.05.

Transcriptome data from diverse plant tissues, including leaves, aerial stems, and rhizomes, were mapped to the mitogenome in a high-precision mode using the Bowtie2 alignment tool. Subsequently, mitochondria-related differentially expressed genes were identified based on read counts using DESeq2. The principal component analysis revealed that the mitochondrial gene expression patterns of *P. australis* exhibited tissue-specific variations (Figure 8a), which reflected the functional differences of mitochondria in different tissues. A total of 43 out of 69 mitochondrial genes were identified as expressed by RNA-Seq (Table S8), with 16 of these exhibiting significant differential expression among the three tissues (FoldChange > 2, *p* value < 0.05), as illustrated in Figure 8b–d. It is important to note that, given the high degree of similarity between the sequences of tRNA genes in the mitogenome and the chloroplast/nuclear genome, there is a risk of errors in RNAseq analyses. Consequently, these tRNA genes should be disregarded when conducting differential expression analyses. In the aerial stem vs. rhizome groups, the expression of the rrn26 gene was significantly upregulated in rhizomes (Figure 8b). In the leaf vs. rhizome comparison, 6 genes were identified as upregulated and 2 as downregulated. The gene expression of cob, nad2, nad4, rpl2, rps19, and rps3 was significantly downregulated in rhizomes. And the expression of the rrn26 gene was also significantly upregulated in rhizomes (Figure 8c). In the leaf vs. aerial stem group, the expressions of the rps3, rpl2, nad2, nad4, nad7, and atp4 genes were significantly downregulated in the aerial stem (Figure 8d).

### 2.8. Phylogenetic Analysis

**Figure 9:**
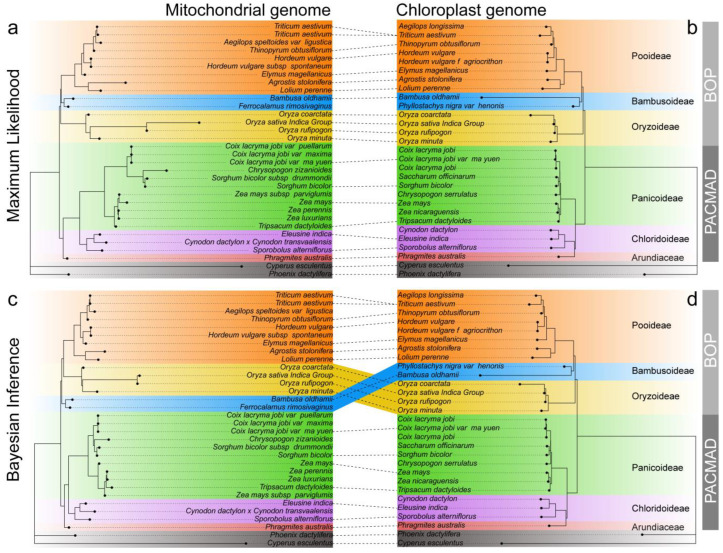
Phylogenetic relationships between *P. australis* and Gramineae. Phylogenetic trees (**a**,**b**) have been constructed using *P. australis* mitochondrial and chloroplast genomes, respectively, based on the maximum likelihood method. Phylogenetic trees (**c**,**d**) have been constructed using *P. australis* mitochondrial and chloroplast genomes, respectively, based on the Bayesian inference method. The topological differences in the phylogenetic trees constructed based on mitochondrial genomes (**left**) and chloroplast genomes (**right**) are shown using phytools (v 2.3-0). Branches connected by dotted lines indicate the matching of different genomic data of the same species in the phylogenetic tree. The use of coloured backgrounds allows for the clear identification of the species belonging to the subfamily to which they belong. Further details pertaining to the phylogenetic tree can be found in Supplementary Figure S6.

We collected 31 mitogenomes and 28 chloroplast genomes published to date in the family Gramineae, based on 33 shared mitochondrial PCGs (*atp1*, *atp4*, *atp6*, *atp8*, *atp9*, *ccmB*, *ccmC*, *ccmFC*, *ccmFN*, *cox1*, *cox2*, *cox3*, *cob*, *matR*, *mttB*, *nad1*, *nad2*, *nad3*, *nad4*, *nad4L*, *nad5*, *nad6*, *nad7*, *nad9*, *rpl2*, *rpl5*, *rpl16*, *rps2*, *rps3*, *rps12*, *rps13*, *rps14*, *rps19*) and 72 shared chloroplast PCGs (*atpA*, *atpB*, *atpE*, *atpH*, *atpI*, *ccsA*, *cemA*, *clpP*, *infA*, *matK*, *ndhA*, *ndhB*, *ndhC*, *ndhD*, *ndhE*, *ndhF*, *ndhG*, *ndhH*, *ndhI*, *ndhJ*, *ndhK*, *petA*, *petB*, *petD*, *petL*, *petN*, *psaA*, *psaB*, *psaC*, *psaI*, *psaJ*, *psbA*, *psbB*, *psbC*, *psbD*, *psbE*, *psbF*, *psbI*, *psbJ*, *psbK*, *psbL*, *psbM*, *psbN*, *psbT*, *psbZ*, *rbcL*, *rpl14*, *rpl16*, *rpl2*, *rpl20*, *rpl22*, *rpl23*, *rpl32*, *rpl33*, *rpl36*, *rpoA*, *rpoB*, *rpoC1*, *rpoC2*, *rps11*, *rps12*, *rps14*, *rps15*, *rps16*, *rps18*, *rps19*, *rps2*, *rps3*, *rps4*, *rps7*, *rps8*, *ycf68*) to construct the phylogenetic tree (Figure 9). In this study, the overwhelming majority of maximum likelihood (ML) bootstrap support values and Bayesian inference (BI) posterior probabilities exhibited high support, indicating a high degree of consistency in the topology of this phylogenetic tree (Figure S6). The results of the phylogenetic analyses indicate that these gramineous species are primarily divided into two large branches, i.e., BOP and PACMAD. All the phylogenetic trees support the conclusion that Arundiaceae and Chloridoideae are sister branches and belong to the PACMAD branch (Figure 9). In the phylogenetic tree constructed based on the ML method, the BOP (including Oryzoideae, Bambusoideae, and Pooideae) branches have consistent topologies in both organelle genomes, both supporting that Pooideae are more closely related to Bambusoideae (Figure 9a,b). However, the BI-constructed phylogenetic tree exhibited discrepancies in the topology of the BOP branch within the two organelle genomes. Specifically, in the mitochondrial genome, Oryzoideae and Pooideae formed sister branches, while in the chloroplast genome, Pooideae and Bambusoideae exhibited a closer relationship (Figure 9c,d).

## 3. Discussion

### 3.1. Genome Assembly and Annotation of the Mitogenome of P. australis

The primary responsibility for photosynthesis and respiration in plants is attributed to chloroplasts and mitochondria with endosymbiotic origins. In comparison to the nuclear genome, organelle-encoded protein genes are more likely to exert a direct regulatory influence on plant biomass accumulation, which is a pivotal factor in enhancing biomass accumulation and crop productivity [25]. *P. australis* is well adapted to harsh environments, such as saline wetlands and desert dunes, and is widely distributed worldwide [2]. The intricacies inherent in the structural variability of the mitochondrial genome, in combination with the inherent limitations of the next-generation sequencing (NGS) technology, have collectively impeded the progress of research in the domain of mitochondrial genome function and evolutionary studies [26]. With the rapid development of the third-generation sequencing (TGS) technology, research on *P. australis* organelle genomes is progressing. Currently, one research result on *P. australis* chloroplast genome [27] and two *P. australis* mitogenome sketch resources have been made public [28,29]. In this study, we devised a pipeline for assembling organelle genomes from genome HiFi sequencing data, utilising a series of publicly available software (Figure 1a). The aforementioned pipeline was employed to successfully assemble a *P. australis* mitogenome with a multibranched structure (Figure 1b) and resolve it into two circular chromosomes (Figure 1c). Furthermore, the *P. australis* mitogenome was demonstrated to possess a high degree of continuity through both long and short data assessment (Figure S2). Additionally, a *P. australis* chloroplast genome with a typical tetrad ring structure was concurrently assembled (Figure S1 and Figure 6). The successful assembly of the *P. australis* mitogenome has extended the comprehension of genetic evolution, functional gene mining, and structural variation of the *P. australis* mitogenome.

A total of 69 genes were identified in the mitogenome of *P. australis*, representing only 6.48% of the total length (Figure 1b, Table S2). It has been demonstrated that three rRNA genes, which are involved in the composition of ribosomes (i.e., *rrn18*, *rrn26*, and *rrn5*), are present in the majority of plant mitogenomes [30,31]. However, some of the published plant mitochondrial genomes are missing some of the rRNA annotations, which may be related to the quality of the mitochondrial genome assembly or the annotation method. For example, *Camellia tianeensis* [32], *Perilla frutescens* [33], *Apostasia fujianica* Y. Li and S. Lan [34], *Fritillaria ussuriensis* Maxim [35], etc. In *P. australis*, the presence of tandemly linked rrn5 and rrn18 genes, along with a rrn26 gene, was identified within two pairs of forward dispersed repeats (Figure 2a). Additionally, we identified two RNA editing sites in this sdh4 gene present in the mitogenome. During the evolutionary process, many important mitochondrial genes in plants were transferred to the nuclear genome to be more finely regulated and protected [36,37,38]. Succinate dehydrogenase (SDH) is a complex of multiple subunits, including SDH1, SDH2, SDH3, and SDH4. It is the only enzyme involved in both the tricarboxylic acid (TCA) cycle and the electron transport chain [39,40]. Each subunit has a specific function within the complex. SDH1 and SDH2 are primarily involved in catalysing the chemical reaction of succinate oxidation, while SDH3 and SDH4 have a primary role in anchoring the complex across membranes and participating in the activity of the electron transport chain, rather than being directly involved in catalysing the reaction [39,40]. In angiosperms, SDH3 and SDH4 typically undergo more frequent loss or transfer to the cytosolic genome during mitogenome evolution [36,41].

Following transfer to the nuclear genome, the rate of nucleotide substitutions in organelle DNA sequences increases significantly [26,38], and this process is controlled by complex gene regulatory mechanisms, including signal peptide sequences and transcription factors [42]. Following its transfer from the mitochondria to the nuclear genome, PaSDH4 did not evolve under significant positive selection (Table S3). Conversely, it remained constrained by negative selection, which suggests that its primary function has maintained the conserved nature of PSDH4 function during evolution. The 29 amino acid constitutive α-helix retained in the *mt_PSDH4* gene may be the smallest structural unit for the anchoring function of the complex that mt_PSDH4 undergoes after evolutionary filtering, whereas other complex functions may be achieved through the transfer of the Nu_PSDH4 subunit into the nuclear genome and undergoing functional expansion or modification of the Nu_PaSDH4 subunit. However, although its primary function has remained largely unchanged, its expression in the nuclear genome may have been adapted for regulatory purposes. The conservation of mt_PSDH4 in *P. australis* mitochondria may result in the emergence of distinctive regulatory mechanisms or enhanced metabolic efficiencies within respiratory and energy metabolic pathways within mitochondria. Furthermore, the *P. australis* mitogenome may represent an intermediate state of gene transfer. This provides a valuable case study for investigating the evolutionary mechanisms underlying mitochondrial gene transfer to the nucleus.

### 3.2. Repetitive Sequences Impact Mitogenome GC Content

Repetitive sequences in mitochondria serve as a valuable source of information for investigating population evolution and molecular markers of species. Repetitive sequences in mitogenomes play a significant role in driving increased genome size, chromosomal structural reorganization, and sequence evolution [43,44]. A total of 129 SSR markers were identified in the mitogenome of *P. australis* in addition to the hexanucleotide repeat type. Single nucleotide repeats and tetranucleotide repeats were the most abundant SSR types in *P. australis* mitochondria (Figure 3a). However, the motif and length polymorphism of these SSR markers exhibited a low level (Table 2). The repeat motifs containing A/T bases in the SSR loci of the mitogenome of *P. australis* were the most prevalent among the different types of SSRs (Figure 3b). Additionally, dispersed and tandem repeat sequences were identified in the *P. australis* mitogenome, exhibiting a lower GC content than AT content (Figure 3c). This may be a consequence of the frequent intermolecular recombination that occurs during the evolutionary process in the mitogenome of *P. australis*, with these repeated sequences also contributing to some extent to the dynamic adjustment of the mitogenome structure or conformation [31,43]. The genomic GC content varies among species and plays an important role in species ecology, distribution, and environmental adaptation [45]. The AT content of the *P. australis* mitogenome was markedly higher than that of CG, a phenomenon that was also observed in the mitogenomes of 31 other gramineous species (Table S1). In conclusion, the higher ratio of A/T bases in the mitogenome sequence is a conserved feature of graminaceous plants. In comparison to the three hydrogen-bond-linked C and G bases, the evolutionary selection of a higher proportion of AT in the *P. australis* genome has resulted in a reduction in the biochemical cost of base synthesis and the energy requirement for genome duplications or conformational changes [46].

### 3.3. Analysis of P. australis Mitochondrial PCGs Ka/Ks and Codon Usage Preferences

Plant mitogenome sequences evolve at low rates of sequence evolution and mutation, but synonymous substitution rates vary dramatically over a relatively small range [47,48]. The overwhelming majority of PCGs in *P. australis* have been subjected to substantial purifying selection (Ka/Ks < 1) throughout evolutionary history. This indicates that these PCGs in *P. australis* are highly conserved among gramineous species (Figure 2).

The role of the codons in the transfer of genetic information from mRNA to protein in organisms is of great significance. The codon usage rates between different species or different genes of the same species gradually produce different degrees of preferential differences, which can be attributed to the process of adaptation and selection of organisms in the long-term evolutionary process [49]. With the exception of rpl16, all PCGs in the *P. australis* mitogenome use ATG as the start codon and preferentially use TAA as the stop codon. There was a notable preference for adenine or thymine (RSCU > 1) in the third base of the *P. australis* mitochondrial PCG codon (Figure 5). This preference for the use of A or T in codons may be a consequence of mitogenome evolution in *P. australis* during long-term adaptation to terrestrial environments. This is analogous to codon preferences observed in higher angiosperms [30,50,51].

### 3.4. Extensive and Frequent Intracellular Gene Transfer (IGT) Events Occur in P. australis

IGT is prevalent in eukaryotic clocks and can increase genome complexity and structural diversity by occurring in a sequential, dynamic manner [24,37,38,52]. Furthermore, it has been shown that IGT can bring about new genes or phenotypes for plants to adapt to environmental changes [11,53,54]. Sequence exchange between mitochondrial and plastid genomes is a common phenomenon observed in Graminaceous plants, including *Oryza sativa* (22,593, 6.30%) [55], *Triticum aestivum* (26,264, 5.80%) [56], *Zea mays* (25,281, 4.40%) [57], *Agrostis stolonifera* (19,114, 3.41%) [58], and *Avena longiglumis* (8207, 1.5%) [59]. A 36,057 bp plastid genome fragment was identified in the mitogenome of *P. australis,* representing 7.20% of the entire mitogenome (Table S5 and Figure 6b). The size and proportion of MTPTs in *P. australis* are notably elevated in comparison to the published mitogenomes of gramineous plants, contributing to the observed expansion in genome size. The genes that migrate from the chloroplasts to the mitochondria gradually become pseudogenes or neogenes, with frequent sequence recombination [60]. Nevertheless, as research into organelle genomes has progressed, it has become evident that certain plastid-derived transfer RNA (tRNA) genes are more conserved than protein-coding genes (PCGs) in mitochondria and retain full functionality [59,61,62]. The sequence of the MTPTs in *P. australis* underwent alterations in polymerisation, translocation, and rearrangement during transfer. However, seven intact tRNA genes of plastid origin were still identified (Figure 6b). The results indicate that there is a high frequency of genetic exchange between the mitochondria and chloroplasts of *P. australis*, and that the functions of some tRNA genes in these transferred fragments show strong conservation. Furthermore, 1,123,956 bp of mitochondrial DNA homologous sequences were identified in the nuclear genome. These mitochondrial-derived sequences have been extensively integrated into all chromosomes of the nuclear genome over an extended period of evolution, which may have significant implications for nuclear genome rearrangement, gene regulation, or pseudogene formation. A comprehensive examination of MTPTs and NUMTs in *P. australis* will yield invaluable insights into the evolutionary history of this species, the evolution of its genome structure, and the interplay between the mitochondrial, chloroplast, and nuclear genomes.

### 3.5. Prediction and Validation of RNA Editing

RNA editing is a phenomenon whereby nucleotides in the coding region of an organelle’s genome are altered post-transcriptionally, resulting in changes to the nucleotide sequence or protein sequence [63,64]. This can contribute to the complexity of the transcriptome at the post-transcriptional stage [65,66]. The mitogenome of *P. australis* exhibits a considerable number of non-synonymous sites, representing 93.51% of all RNA editing sites (Figure 7b). As plant mitogenomes have evolved over time, RNA editing sites have demonstrated a gradual preference [30,67,68]. There is a strong leucine tendency (42.39% of all) for RNA editing of amino acids in key proteins (e.g., ATP synthase subunits, NADH dehydrogenase subunits, cytochrome c oxidase subunits, etc.) in *P. australis* mitochondria (Figure 7b). Leucine, as a hydrophobic amino acid, is involved in the formation and stabilisation of secondary structures such as α-helices or β-folds, promoting protein folding and structural stability [69,70]. The leucine-propensity RNA editing events observed in *P. australis* may facilitate the integration of electron transport genes into the inner mitochondrial membrane, thereby enhancing their functionality. This process may also assist *P. australis* in preserving the stability of mitochondrial proteins in challenging environmental conditions. In addition, *P. australis* generates stop codons or start codons by RNA editing in important genes (e.g., atp6, atp9, and ccmFC), which ensures more flexible regulation of key enzymes in the respiratory chain and ATP synthesis process under specific environmental conditions, further increasing *P. australis*’s ability to respond to environmental changes. The distribution and editing efficiency of RNA editing have been recently shown to be tissue- and developmental stage-specific [64,66]. Furthermore, RNA editing events have been demonstrated to play a pivotal role in plant response to a variety of environmental stresses [71,72,73,74,75]. For instance, RNA editing efficiencies in matK, accD, atpB, rpoC2, and petA in the cucumber chloroplast genome have been shown to be significantly increased by high-temperature stress, and RNA editing efficiencies in rpoB, psaA, rbcL, and accD were significantly reduced by low-temperature stress [72]. Salt stress also significantly increased the efficiencies of multiple RNA editing sites in the barley mitogenome in nad3, nad7, and ccmfn transcripts in multiple RNA editing sites [73,74,75]. Based on transcriptomic data from different tissues, we confirmed the accuracy of these RNA editing sites and, also, showed significant tissue-specific differences (Figure S5). These identified and confirmed RNA editing sites in the *P. australis* mitogenome provide important clues for probing the adaptive mechanisms and gene expression regulation of plants to environmental stresses and lay the data foundation for resolving the environmental adaptive capacity of *P. australis* such as salinity and hypoxia tolerance.

### 3.6. Analysis of Tissue-Specific Differential Expression Gene of P. australis Mitogenomes

The complex rhizome system of *P. australis* is closely related to its perennial plant characteristics, which provide *P. australis* with a strong nutrient storage capacity, lateral expansion, stress resistance, and regeneration [76,77,78]. However, because rhizomes act as underground organs, they are often directly exposed to harsh environments such as salinity, flooding, heavy metals, or low oxygen during long-term survival and growth [79,80]. Recent evidence suggests that plants may be involved in adversity response by regulating mitochondrial and chloroplast gene expression or protein transfer [81,82,83,84,85]. Compared to leaves, *P. australis* rhizomes reduced the expression of core protein genes (cob, nad2, nad4) encoding the mitochondrial respiratory chain (Figure 8b). This not only reduced the energy consumption of the electron transport chain and respiratory activity in the mitochondria but also alleviated the accumulation of reactive oxygen species (ROS) due to oxygen deprivation. Unlike aerial stems or leaves, which grow and reproduce rapidly, the main function of *P. australis* rhizomes is to store nutrients (e.g., starch) and maintain regenerative functions. The downregulation of mitochondrial ribosomal protein-coding genes (rpl2, rps19, and rps3) in *P. australis* rhizomes may reflect that *P. australis* rhizomes have been maintained in a state of low metabolism for a long time, reducing the demand for protein synthesis (Figure 8c,d). Taken together, we hypothesise that the downregulation of these genes in *P. australis* rhizomes may be an adaptive strategy for survival in chronically low-oxygen soil environments, prioritising energy storage and conservation and reducing high metabolic activity. It is worth noting that the mechanisms regulating the tissue-specific expression of these mitochondrial genes need to be refined to provide further evidence, but our results, nevertheless, provide new insights into adaptive evolution in *P. australis* in response to complex habitats.

### 3.7. Phylogenetic Analyses

Maternally inherited plant mitogenomes are highly conserved and have very low recombination rates, making them valuable for population genetic and phylogenetic studies [86,87]. Here, for the first time, we used *P. australis* mitogenome sequences to construct a phylogenetic tree of 31 graminaceous species, which we confirmed using the chloroplast genomes of 28 species (Figure 9). Our results support the taxonomic relationship between the graminoid BOP and PACMAD and demonstrate the evolutionary affinity of *P. australis* with the Chloridoideae. This is consistent with the evolutionary relationships of the nuclear genome [28,88,89]. Notably, there were topological differences in the BOP branches in the phylogenetic trees constructed based on the BI method (Figure 9c,d), which could be caused by several factors. The structural reorganisation and evolutionary history of mitogenomes are usually more complex than that of chloroplast genomes. At the same time, the high mutation rate and recombination frequency of mitogenomes may also lead to complex phylogenetic relationships [26,90]. Moreover, the sensitivity of the BI method to a priori assumptions and the long branch attraction effect (LBA) may be an important factor contributing to the topological differences between the mitochondrial genome tree and the chloroplast genome tree. This study revealed different phylogenetic relationships among gramineae in mitochondrial and chloroplast genomes by different methods, demonstrating the complexity of model selection and data characterisation in species evolutionary analysis. Subsequent research endeavours should incorporate a more substantial array of genomic data to further substantiate the evolutionary relationships among Gramineae species and furnish a more exhaustive and profound perspective on the historical evolution of species.

## 4. Materials and Methods

### 4.1. Sample Collection and Sequencing Data

This study’s organelle genome read data were derived from sequencing data from a previous *P. australis* genome project (PRJNA1055898) in our laboratory [88]. This project’s *P. australis* strain material (*Phragmites australis* (Cav.) var. Cuiplus) was conserved at the Capital Normal University Reed Planting Sample Site.

### 4.2. Genome Assembly and Annotation

a. Sequencing data quality control: 32.6 Gbp HiFi reads were obtained from *P. australis* genome PacBio HIFI sequencing raw read data using pbccs [88] software; parameters: --min-rq 0.99 --min-passes 3. SOAPnuke [91] filtered BGI T7 short raw reads with connectors and low-quality reads to obtain clean reads; parameters: -lowQual = 20, -nRate = 0.005, -qualRate = 0.5.

b. Assembly of organelle genome master graphs (MGs): First, the organelle genome was assembled with MGs using autoMito from the PMAT (v1.5.3) toolkit [92] with the following parameters: -st hifi -g 849m -fc 0.2. The assembly result was then visualised using Bandage (v0.9.0) [93], and all mitogenome MG contig sequences were exported.

c. Contig sequence filtering: A subject sequence database was constructed using 30 Gramineae mitogenomes, and the contig sequences of MGs were used as query sequences in BLASTn (v 2.12.0) [94] to remove all contig sequences that were not aligned with the subject sequences.

d. Acquisition of reads for mitogenome assembly: first, HiFi long reads were aligned to filtered contig sequences using minimap2 (2.24-r1122) [95], retaining sequences with alignment coverage greater than or equal to 0.7 and alignment length greater than or equal to 1000 bp of alignments, and further retaining long reads with length greater than or equal to 3000 bp using seqkit (v 2.1.0) [96]. Clean short reads were then mapped to filtered contigs using BWA (0.7.18-r1243-dirty) [97], and all mapped reads were retained using SAMtools (v 1.13) [98]. Finally, long- and short-read data were obtained separately to assemble the mitogenome.

e. DBS parsing: To parse double bifurcating structures (DBS) in MGs, we performed a complete mitogenome assembly of *P. australis* using Unicycler (v 0.5.1) [99] in combination with filtered long reads and short reads. First, the short reads were assembled into a preliminary assembly graph using the SPAdes (v3.14.0) software built into Unicycler with the following parameters: -k 27 53 71 87 99 111 119 127. Next, the preliminary assembly graph was parsed using the built-in miniasm and Racon to determine the DBS structure by comparing the long reads. Graph with the DBS structure. Finally, we obtained two complete ring chromosomes of the *P. australis* mitogenome.

f. Assessment of mitogenome integrity and continuity: The integrity and continuity of the mitogenome were assessed by mapping the long and short reads used to assemble the mitogenome onto two circular chromosomes and calculating the sequencing depth and read coverage. For methods, see dx.doi.org/10.17504/protocols.io.4r3l27jkxg1y/v1, accessed on 22 September 2024.

The mitogenome annotation identified two circular mitochondrial chromosome sequences, which were analysed using IPMGA (http://www.1kmpg.cn/ipmga/, accessed on 24 July 2024). The tRNA annotations were then subjected to further validation and adjustment using tRNAscan-SE v. 2.0 [100]. The annotation results were manually proofread using Geneious Prime 2024.0.5 (https://www.geneious.com, accessed on 14 April 2024). Finally, the two circular mitogenomes were visualised using OGView (http://www.1kmpg.cn/ogview, accessed on 26 July 2024). The assembled chloroplast MGs genome was disassembled into a circular tetrameric structure using Bandage (v0.9.0) based on the *P. australis* chloroplast reference genome (NC_022958.1). Subsequently, the obtained circular chloroplast genome was annotated using CPGAVAS2 [101], and the annotations were manually proofread in Geneious Prime 2024.0.5 (https://www.geneious.com, accessed on 14 April 2024). The visualisation of the chloroplast gene structure was performed using CPGView.

### 4.3. Identification of the Succinate Dehydrogenase Gene Family

To identify members of the succinate dehydrogenase (SDH) family within the nuclear genome of *P. australis*, the sequences of SDH family proteins were downloaded from 13 Arabidopsis thaliana and 14 Oryza sativa. Subsequently, a search was conducted in the *P. australis* genome (PaCui.No1) [88] using the BLAST tool to identify sequence homology to Arabidopsis and rice SDH proteins. Subsequently, the sequences identified in the BLAST results were screened using the CDD (Conserved Domain Database) to verify their functional relevance. Subsequently, a multiple sequence alignment was conducted using the MAFFT (v 7.490) [102], and a maximum likelihood phylogenetic tree was constructed using the IQ-TREE (v 2.3.6) [103] with a bootstrap value of 1000 and the WAG + R2 model. The evolutionary tree was visualised using the Interactive Tree of Life (iTOL) platform (https://itol.embl.de/, accessed on 18 July 2024). Protein structures were predicted using the AlphaFold server (https://alphafoldserver.com/, accessed on 15 October 2024).

### 4.4. Identification of Repeat Sequences

Simple sequence repeats (SSRs) in the mitogenome were identified using MISA (v 2.1) [104]. The minimum number of single nucleotides, dinucleotides, trinucleotides, tetranucleotides, pentanucleotides, and hexanucleotides was set to 10, 5, 4, 3, 3, and 3, respectively. Tandem repeat sequences in the mitogenome were detected using TRF (v 4.09) [105] with the parameters of ‘2 7 7 80 10 50 500 -f -d -m’. Scattered repetitive sequences in the mitogenome were detected using REPuter (https://bibiserv.cebitec.uni-bielefeld.de/reputer/, accessed on 28 July 2024). The density and position of these repetitive sequences on the mitogenome were plotted using the Circos [106].

### 4.5. Analysis of Synonymous and Nonsynonymous Substitution Rates

The Ka/Ks ratio of PCGs in the mitogenome of *P. australis* was analysed using the mitogenomes of 32 Gramineae species as a reference. The following accession numbers were used in the analysis: AP008982, AP013107, EU365401, JN120789, JX999996, MG429050, MK175054, MN127966, MN127974, MT471097, MT471098, MT4710 99, MT471321, MZ506736, NC_007886, NC_007982, NC_008331, NC_008332, NC_008333, NC_008360, NC_008362, NC_013816, NC_ Additionally, the following reference species protein sequences were identified: 016740, NC_029816, NC_036024, NC_040989, NC_056367, NC_058697, OK037503-OK037504, OK120846, OQ086977, and OQ695465. The reference species protein sequences were best matched to *P. australis* protein sequences using BLASTn (v2.10.1) to obtain homologous protein sequences. Subsequently, the shared homologous protein sequences were compared using MAFFT (v 7.490), and Ka/Ks values of *P. australis* mitochondrial genes with homologous genes in other species were calculated using Ka/Ks_Calculator 2.0 [107].

### 4.6. Codon Usage Analysis

The sequences of all protein-coding genes (PCGs) in the mitogenome of *P. australis* were extracted using PhyloSuite (v 1.2.3) [108], and the relative synonymous codon usage (RSCU) was calculated. A value of RSCU = 1 indicates unbiased codon usage. In contrast, a value of RSCU < 1 suggests that the actual frequency of the codon in question is lower than that of other synonymous codons. Conversely, a value of RSCU > 1 indicates that the actual frequency of the codon is higher than that of other synonymous codons.

### 4.7. Identification of Mitochondrial Plastid DNA Segments (MTPTs) and Nuclear Mitochondrial DNA Segments (NUMTs)

To identify the sequences of MTPTs and NUMTs in the mitogenome of *P. australis*, the mitogenome sequences of *P. australis* were subjected to Reciprocal Best Hit BLAST analysis with the chloroplast genome sequences and the nuclear genome sequences assembled in the present study (PaCui.No1), respectively. The E-value was set at 1 × 10^−5^, and BLASTn was used for this purpose. The sequences were visualised using TBtools (v 2.128) [109] in the Circos program, which visualised the MTPT and NUMT sequences.

### 4.8. RNA Editing Site Prediction and Mitochondrial Gene Expression Analysis

Deepred-Mt can predict C to U editing sites in angiosperm mitochondrial RNA based on a deep neural network approach. The Deepred-Mt [110] tool was employed to predict RNA editing events in the mitogenome of *P. australis*, with a prediction probability value exceeding 0.9 within the protein-coding region retained. To validate the accuracy of the predicted RNA editing sites, nine transcriptome datasets from *P. australis* leaves, aerial stems, and rhizomes were used to identify RNA editing sites. Firstly, the filtered short reads genomic data were mapped to the *P. australis* mitochondrial PCG sequences using BWA (0.7.18-r1243-dirty) [97], and the SNP sites in the *P. australis* mitogenome were identified using BCFtools (v 1.13) [98]. The SNPs were then filtered based on the ‘DP < 30, AF > 0.1’ criteria. Subsequently, the RNAseq data were mapped to the *P. australis* mitochondrial PCG sequences, and the RNA editing sites were identified using REDItools (v 2.0) [111] with the following parameters: -S, -c 30, -m 0.1, -p 0.05. Ultimately, the SNPs within the PCG region of the *P. australis* mitogenome were filtered out to obtain the final RNA editing site information.

To investigate the differential expression patterns of *P. australis* mitochondrial genes in different tissues, nine transcriptome datasets comprising leaves, aerial stems, and rhizomes of the same *P. australis* plant were used. This was performed to identify mitochondrial differentially expressed genes (mtDEGs). The initial step involved mapping bipartite sequencing RNA-seq reads onto mitogenome PCG sequences, utilising the Bowtie2 software (version 2.4.4) [112] by applying rigorous parameters, including: --very-sensitive --no-unal --no-mixed --no-discordant -k 1 --score-min L,0,-0.6 -N 1. Subsequently, mitochondrial genes were subjected to differential expression analysis using DESeq2 (v 1.44.0) [113], and genes with a P-value of less than 0.05 and a fold change greater than 2 were designated as mitochondrial differential expression genes in this study. Finally, principal component analysis and volcano plotting were performed using FactoMineR [114] and ggplot2 [115].

### 4.9. Phylogenetic Analysis

The initial step involved retrieving and downloading 31 mitogenomes and 28 chloroplast genomes of gramineous species from the NCBI database to construct a phylogenetic tree. The outgroups included Phoenix dactylifera and Cyperus esculentus (Figure 9). Next, based on the PCG genes of *P. australis* mitogenomes, the PCG genes shared in these genomes were extracted using PhyloSuite (v 1.2.3) [108]. Subsequently, multiple sequence alignment was performed using MAFFT (v 7.490), and low-quality aligned sites in the file were trimmed using trimAl (v1.2rev57) [116] with parameter ‘-automated1’ comparison. The trimmed PCG sequences were ligated and used to construct a phylogenetic tree. Ultimately, the most optimal nucleoside substitution models were identified utilising ModelFinder (v2.2.0) [117], and phylogenetic analyses were conducted employing IQ-TREE (v 2.3.6) and MrBayes (v3.2.7a) [118]. The final results of the phylogenetic analyses were visualised using the Interactive Tree of Life (iTOL) online tool (https://itol.embl.de/, accessed on 24 July 2024).

## 5. Conclusions

In this study, the mitogenome with a multibranched structure was filtered and assembled from *P. australis* genome sequencing data and parsed to form two circular chromosomes using long-read data. A total of 69 genes were annotated to this *P. australis* mitogenome, including a succinate dehydrogenase subunit 4 gene whose protein sequence was streamlined to only 29 amino acids. The mitogenome of *P. australis* contains a high proportion of A/T bases in its repeat sequences. Furthermore, the codons of these evolutionarily highly conserved PCGs also demonstrate a strong A/T base preference. These sequence features are conserved in the gramineous mitogenome, which may reduce the biochemical and energetic costs associated with base synthesis and repetitive sequence-mediated structural reorganisation. Extensive and frequent intracellular gene transfer events have occurred in the mitogenome of *P. australis*, which provides new genetic and phenotypic evidence for plant adaptation to environmental change. RNA editing displays a high degree of diversity and tissue specificity in key mitochondrial genes of *P. australis*, which serve as a mechanism that enhances the capacity of *P. australis* to regulate respiratory key genes in a more direct and efficient manner in response to environmental changes. The tissue-specific differential expression analysis of mitochondrial genes in *P. australis* has revealed a low-energy and low-metabolism expression pattern in *P. australis* rhizomes, which are shaped for efficient storage of nutrients in a long-term low-oxygen soil environment. Furthermore, comprehensive phylogenetic analyses of organelle genomes in this study support the taxonomic relationship between BOP and PACMAD in Gramineae and reveal close affinities between Arundiaceae and Chloridoideae. In conclusion, this study contributes to the comprehension of genetic evolution and gene expression in the mitogenome of *P. australis* and offers a significant case study for investigating the evolutionary processes of intracellular gene transfer.

## Figures and Tables

**Table 1:** Genes predicted in the *P. australis* mitogenome.

Group of Genes		Name of Genes
Protein coding genes (PCGs)	ATP synthase	*atp1*, *atp4*, *atp6 (×2)*, *atp8 (×2)*, *atp9*
	NADH dehydrogenase	*nad1*, *nad2*, *nad3*, *nad4*, *nad4L*, *nad5*, *nad6*, *nad7*, *nad9*
	Cytochrome c biogenesis	*cob*
	Ubiquinol cytochrome c reductase	*ccmB*, *ccmC*, *ccmFC*, *ccmFN*
	Cytochrome c oxidase	*cox1*, *cox2*, *cox3*
	Maturases	*matR*
	Transport membrane protein	*mttB*
	Large subunit of the ribosome	*rpl5*, *rpl16*, *rpl2*
	Small subunit of ribosome	*rps2*, *rps3*, *rps12*, *rps13*, *rps14*, *rps19*
	Succinate dehydrogenase	*sdh4*
Ribosomal RNA	Ribosomal RNAs	*rrn5 (×2)*, *rrn18 (×2)*, *rrn26 (×2)*
Transfer RNA	Transfer RNAs	*trnC-GCA*, *trnD-GUC (×2)*, *trnE-UUC*, *trnfM-CAU (×2)*, *trnH-GUG*, *trnI-CAU (×2)*, *trnL-CAA*, *trnN-GUU*, *trnP-UGG (×2)*, *trnQ-UUG*, *trnR-UCU*, *trnS-GGA (×2)*, *trnS-UGA*, *trnV-UAC*, *trnF-GAA (×2)*, *trnK-UUU (×2)*, *trnM-CAU*, *trnS-GCU*, *trnW-CCA*, *trnY-GUA*

**Table 2:** Frequency of identified SSR motifs in the *P. australis* mitogenome.

Repeats Type	Number of Repeats												Total	Proportion (%)
	3	4	5	6	7	8	9	10	11	12	13	14		
Monomeric	-	-	-	-	-	-	-	37	7	2	3	1	50	38.76
Dimeric	-	-	17	4	1	-	-	-	-	-	-	-	22	17.05
Trimeric		13	1	1	1	-	-	-	-	-	-	-	17	13.18
Tetrameric	35	1	-	-	-	-	-	-	-	-	-	-	37	28.68
Pentameric	2	1	-	-	-	-	-	-	-	-	-	-	3	2.33
total	38	16	18	5	2	0	0	37	7	2	3	1	129	100.00

## Data Availability

Whole genome and transcriptome raw sequencing data used in this study have been deposited at the National Center for Biotechnology Information (NCBI) under accession number PRJNA1055898. The *P. australis* mitogenome assembly has been deposited in the NCBI database with accession Nos: PQ456902-PQ456903.

## Supplementary Materials

The following supporting information can be downloaded at: https://www.mdpi.com/article/10.3390/ijms26020546/s1.

## References

[1] Cahoon D.R., McKee K.L. (2021). How Plants Influence Resilience of Salt Marsh and Mangrove Wetlands to Sea-Level Rise. Estuaries Coasts.

[2] Cui J.P., Qiu T.H. (2023). De novo full-length transcriptome analysis of two ecotypes of *Phragmites australis* (swamp reed and dune reed) provides new insights into the transcriptomic complexity of dune reed and its long-term adaptation to desert environments. BMC Genom..

[3] Hua W.T., Hu W.Q. (2023). Identification of microbial consortia for sustainable disposal of constructed wetland reed litter wastes. Environ. Sci. Pollut. Res..

[4] Wang Y.N., Cui X.J. (2021). Cyclic utilization of reed litters to enhance nitrogen removal efficiency in simulated estuarine wetland. Environ. Sci. Pollut. Res..

[5] Cui M.X., Fang Z. (2022). *Phragmites rhizoma* polysaccharide-based nanocarriers for synergistic treatment of ulcerative colitis. Int. J. Biol. Macromol..

[6] Ren Y., Cui G.D. (2022). Traditional Uses, Phytochemistry, Pharmacology and Toxicology of *Rhizoma phragmitis*: A Narrative Review. Chin. J. Integr. Med..

[7] Zhou R.M., Cui M.X., Wang Y., Zhang M., Li F., Liu K. (2020). Isolation, structure identification and anti-inflammatory activity of a polysaccharide from *Phragmites rhizoma*. Int. J. Biol. Macromol..

[8] Brownfield L., Yi J. (2015). Organelles maintain spindle position in plant meiosis. Nat. Commun..

[9] Kubo T., Newton K.J. (2008). Angiosperm mitochondrial genomes and mutations. Mitochondrion.

[10] Osiewacz H.D., Brust D. (2010). Mitochondrial pathways governing stress resistance, life, and death in the fungal aging model *Podospora anserina*. Ann. N. Y. Acad. Sci..

[11] Gualberto J.M., Mileshina D. (2014). The plant mitochondrial genome: Dynamics and maintenance. Biochimie.

[12] Cheng N., Lo Y.S. (2017). Correlation between mtDNA complexity and mtDNA replication mode in developing cotyledon mitochondria during mung bean seed germination. New Phytol..

[13] Wu Z.Q., Cuthbert J.M. (2015). The massive mitochondrial genome of the angiosperm is evolving by gain or loss of entire chromosomes. Proc. Natl. Acad. Sci. USA.

[14] Komatsu S., Yamamoto A. (2011). Comprehensive Analysis of Mitochondria in Roots and Hypocotyls of Soybean under Flooding Stress using Proteomics and Metabolomics Techniques. J. Proteome Res..

[15] Qin G.Z., Meng X.H. (2009). Oxidative Damage of Mitochondrial Proteins Contributes to Fruit Senescence: A Redox Proteomics Analysis. J. Proteome Res..

[16] Tan Y.F., Millar A.H. (2012). Components of Mitochondrial Oxidative Phosphorylation Vary in Abundance Following Exposure to Cold and Chemical Stresses. J. Proteome Res..

[17] Colombatti F., Mencia R. (2019). The mitochondrial oxidation resistance protein AtOXR2 increases plant biomass and tolerance to oxidative stress. J. Exp. Bot..

[18] Wang J.Y., Xu G.J. (2022). Mitochondrial functions in plant immunity. Trends Plant Sci..

[19] Yang Y., Zhao Y. (2022). A mitochondrial RNA processing protein mediates plant immunity to a broad spectrum of pathogens by modulating the mitochondrial oxidative burst. Plant Cell.

[20] Han F., Qu Y. (2022). Assembly and comparative analysis of the complete mitochondrial genome of *Salix wilsonii* using PacBio HiFi sequencing. Front. Plant Sci..

[21] Sun Y.Q., Shang L.G. (2022). Twenty years of plant genome sequencing: Achievements and challenges. Trends Plant Sci..

[22] Wang Z., Wang R. (2024). Comparative analysis of mitochondrial genomes of invasive weed *Mikania micrantha* and its indigenous congener *Mikania cordata*. Int. J. Biol. Macromol..

[23] Plotkin J.B., Kudla G. (2011). Synonymous but not the same: The causes and consequences of codon bias. Nat. Rev. Genet..

[24] Wei W., Schon K.R. (2022). Nuclear-embedded mitochondrial DNA sequences in 66,083 human genomes. Nature.

[25] Raven J.A. (2015). Implications of mutation of organelle genomes for organelle function and evolution. J. Exp. Bot..

[26] Wang J., Kan S. (2024). Plant organellar genomes: Much done, much more to do. Trends Plant Sci..

[27] Qiu T.H., Cui S.X. (2021). Evolutionary analysis for Phragmites ecotypes based on full-length plastomes. Aquat. Bot..

[28] Wang C., Liu L. (2024). Chromosome-level genome assemblies reveal genome evolution of an invasive plant *Phragmites australis*. Commun. Biol..

[29] Christenhusz M.J.M., Fay M.F. (2024). The genome sequence of common reed, *Phragmites australis* (Cav.) Steud. (Poaceae). Wellcome Open Res..

[30] Cheng Y., He X.X. (2011). Assembly and comparative analysis of the complete mitochondrial genome of *Suaeda glauca*. BMC Genom..

[31] Zhou P., Zhang Q. (2023). Assembly and comparative analysis of the complete mitochondrial genome of *Ilex metabaptista* (Aquifoliaceae), a Chinese endemic species with a narrow distribution. BMC Plant Biol..

[32] Li Z., Ran Z., Xiao X., Yan C., Xu J., Tang M., An M. (2024). Comparative analysis of the whole mitochondrial genomes of four species in sect. *Chrysantha* (*Camellia* L.), endemic taxa in China. BMC Plant Biol..

[33] Wang R., Luo Y., Lan Z., Qiu D. (2024). Insights into structure, codon usage, repeats, and RNA editing of the complete mitochondrial genome of *Perilla frutescens* (Lamiaceae). Sci. Rep..

[34] Zheng Q., Luo X., Huang Y., Ke S.-J., Liu Z.-J. (2024). The Complete Mitogenome of *Apostasia fujianica* Y.Li & S.Lan and Comparative Analysis of Mitogenomes across Orchidaceae. Int. J. Mol. Sci..

[35] Xie P., Wu J., Lu M., Tian T., Wang D., Luo Z., Yang D., Li L., Yang X., Liu D. (2024). Assembly and comparative analysis of the complete mitochondrial genome of *Fritillaria ussuriensis* Maxim. (Liliales: Liliaceae), an endangered medicinal plant. BMC Genom..

[36] Adams K.L., Palmer J.D. (2003). Evolution of mitochondrial gene content: Gene loss and transfer to the nucleus. Mol. Phylogenet Evol..

[37] Archibald J.M. (2011). Origin of eukaryotic cells: 40 years on. Symbiosis.

[38] Chen Y.M., Guo Y.W. (2023). Pangenome-based trajectories of intracellular gene transfers in Poaceae unveil high cumulation in Triticeae. Plant Physiol..

[39] Choi C., Liu Z. (2006). Evolutionary transfers of mitochondrial genes to the nucleus in the *Populus* lineage and coexpression of nuclear and mitochondrial *Sdh4* genes. New Phytol..

[40] Huang S.B., Millar A.H. (2013). Succinate dehydrogenase: The complex roles of a simple enzyme. Curr. Opin. Plant Biol..

[41] Hall N.D., Zhang H., Mower J.P., McElroy J.S., Goertzen L.R. (2020). The Mitochondrial Genome of Eleusine indica and Characterization of Gene Content within Poaceae. Genome Biol. Evol..

[42] Zhu D., Li X.Y. (2022). Mitochondrial-to-nuclear communication in aging: An epigenetic perspective. Trends Biochem. Sci..

[43] Guo W.H., Grewe F. (2016). Ginkgo and Welwitschia Mitogenomes Reveal Extreme Contrasts in Gymnosperm Mitochondrial Evolution. Mol. Biol. Evol..

[44] Song Y., Du X.R. (2023). Assembly and analysis of the complete mitochondrial genome of *Forsythia suspensa* (Thunb.) Vahl. BMC Genom..

[45] Mann S., Chen Y.P.P. (2010). Bacterial genomic G+C composition-eliciting environmental adaptation. Genomics.

[46] Smarda P., Bures P. (2014). Ecological and evolutionary significance of genomic GC content diversity in monocots. Proc. Natl. Acad. Sci. USA.

[47] Christensen A.C. (2021). Plant Mitochondria are a Riddle Wrapped in a Mystery Inside an Enigma. J. Mol. Evol..

[48] Drouin G., Daoud H. (2008). Relative rates of synonymous substitutions in the mitochondrial, chloroplast and nuclear genomes of seed plants. Mol. Phylogenet Evol..

[49] Parvathy S.T., Udayasuriyan V. (2022). Codon usage bias. Mol. Biol. Rep..

[50] Qiao Y.G., Zhang X.R. (2022). Assembly and comparative analysis of the complete mitochondrial genome of *Bupleurum chinense* DC. BMC Genom..

[51] Yang J., Wariss H.M. (2019). De novo genome assembly of the endangered *Acer yangbiense*, a plant species with extremely small populations endemic to Yunnan, China. Gigascience.

[52] Wu Z.Q., Liao X.Z. (2022). Genomic architectural variation of plant mitochondria-A review of multichromosomal structuring. J. Syst. Evol..

[53] Chen J.H., Chen S.T., He N.Y., Wang Q.L., Zhao Y., Gao W., Guo F.Q. (2020). Nuclear-encoded synthesis of the D1 subunit of photosystem II increases photosynthetic efficiency and crop yield. Nat. Plants.

[54] Ma J.C., Wang S.H., Zhu X.J., Sun G.L., Chang G.X., Li L.H., Hu X.Y., Zhang S.Z., Zhou Y., Song C.P. (2022). Major episodes of horizontal gene transfer drove the evolution of land plants. Mol. Plant..

[55] Notsu Y., Masood S., Nishikawa T., Kubo N., Akiduki G., Nakazono M., Hirai A., Kadowaki K. (2002). The complete sequence of the rice (*Oryza sativa* L.) mitochondrial genome: Frequent DNA sequence acquisition and loss during the evolution of flowering plants. Mol. Genet. Genom..

[56] Ogihara Y., Yamazaki Y., Murai K., Kanno A., Terachi T., Shiina T., Miyashita N., Nasuda S., Nakamura C., Mori N. (2005). Structural dynamics of cereal mitochondrial genomes as revealed by complete nucleotide sequencing of the wheat mitochondrial genome. Nucleic Acids Res..

[57] Clifton S.W., Minx P., Fauron C.M., Gibson M., Allen J.O., Sun H., Thompson M., Barbazuk W.B., Kanuganti S., Tayloe C. (2004). Sequence and comparative analysis of the maize NB mitochondrial genome. Plant Physiol..

[58] Li J.X., Chen Y.L., Liu Y.L., Wang C., Li L., Chao Y.H. (2023). Complete mitochondrial genome of *Agrostis stolonifera*: Insights into structure, Codon usage, repeats, and RNA editing. BMC Genom..

[59] Liu Q., Yuan H., Xu J., Cui D., Xiong G., Schwarzacher T., Heslop-Harrison J.S. (2023). The mitochondrial genome of the diploid oat *Avena longiglumis*. BMC Plant Biol..

[60] Anderson B.M., Krause K., Petersen G. (2021). Mitochondrial genomes of two parasitic *Cuscuta* species lack clear evidence of horizontal gene transfer and retain unusually fragmented ccmF(C) genes. BMC Genom..

[61] Bergthorsson U., Adams K.L., Thomason B., Palmer J.D. (2003). Widespread horizontal transfer of mitochondrial genes in flowering plants. Nature.

[62] Joyce P.B., Gray M.W. (1989). Chloroplast-like transfer RNA genes expressed in wheat mitochondria. Nucleic Acids Res..

[63] Gray M.W. (2009). RNA editing in plant mitochondria: 20 years later. IUBMB Life.

[64] Miyata Y., Sugita M. (2004). Tissue- and stage-specific RNA editing of rps 14 transcripts in moss (*Physcomitrella patens*) chloroplasts. J. Plant Physiol..

[65] Duan Y.E., Ma L., Liu J.Y., Liu X.Z., Song F., Tian L., Cai W.Z., Li H. (2024). The first A-to-I RNA editome of hemipteran species *Coridius chinensis* reveals overrepresented recoding and prevalent intron editing in early-diverging insects. Cell Mol. Life Sci..

[66] Fang J., Jiang X.H., Wang T.F., Zhang X.J., Zhang A.D. (2021). Tissue-specificity of RNA editing in plant: Analysis of transcripts from three tobacco (*Nicotiana tabacum*) varieties. Plant Biotechnol. Rep..

[67] Knoop V. (2023). C-to-U and U-to-C: RNA editing in plant organelles and beyond. J. Exp. Bot..

[68] Xu Y., Dong Y., Cheng W., Wu K., Gao H., Liu L., Xu L., Gong B. (2022). Characterization and phylogenetic analysis of the complete mitochondrial genome sequence of *Diospyros oleifera*, the first representative from the family Ebenaceae. Heliyon.

[69] Zavrtanik U., Medved T., Puric S., Vranken W., Lah J., Hadzi S. (2024). Leucine Motifs Stabilize Residual Helical Structure in Disordered Proteins. J. Mol. Biol..

[70] Zhu B.Y., Zhou N.E., Kay C.M., Hodges R.S. (1993). Packing and hydrophobicity effects on protein folding and stability: Effects of beta-branched amino acids, valine and isoleucine, on the formation and stability of two-stranded alpha-helical coiled coils/leucine zippers. Protein Sci..

[71] Hu Y.X., Huang A., Li Y., Molloy D.P., Huang C. (2024). Emerging roles of the C-to-U RNA editing in plant stress responses. Plant Sci..

[72] Xia L., Wang H., Zhao X., Obel H.O., Yu X., Lou Q., Chen J., Cheng C. (2023). Chloroplast Pan-Genomes and Comparative Transcriptomics Reveal Genetic Variation and Temperature Adaptation in the Cucumber. Int. J. Mol. Sci..

[73] Ramadan A.M. (2020). Salinity effects on nad3 gene RNA editing of wild barley mitochondria. Mol. Biol. Rep..

[74] Ramadan A.M., Said O.A.M., Abushady A.M. (2022). Salinity stress reveals three types of RNA editing sites in mitochondrial Nad7 gene of wild barley both in silico and in qRT-PCR experiments. Theor. Exp. Plant Physiol..

[75] Ramadan A., Alnufaei A.A., Fiaz S., Khan T.K., Hassan S.M. (2023). Effect of salinity on ccmfn gene RNA editing of mitochondria in wild barley and uncommon types of RNA editing. Funct. Integr. Genom..

[76] Gao J., Guan B., Ge M., Eller F., Yu J., Wang X., Zuo J. (2022). Can allelopathy of *Phragmites australis* extracts aggravate the effects of salt stress on the seed germination of Suaeda salsa?. Front. Plant Sci..

[77] He R., Kim M.J., Nelson W., Balbuena T.S., Kim R., Kramer R., Crow J.A., May G.D., Thelen J.J., Soderlund C.A. (2012). Next-generation sequencing-based transcriptomic and proteomic analysis of the common reed, *Phragmites australis* (Poaceae), reveals genes involved in invasiveness and rhizome specificity. Am. J. Bot..

[78] Williams J., Lambert A.M., Long R., Saltonstall K. (2019). Does hybrid *Phragmites australis* differ from native and introduced lineages in reproductive, genetic, and morphological traits?. Am. J. Bot..

[79] Cui M.Y., Du Z.X., Li X.Y., Chen J.Z. (2022). Physiological and ecological characteristics and reproductive responses of *Phragmites australis* to dry-wet conditions in inland saline marshes of Northeast China. PeerJ.

[80] Wu J., Gao T., Zhao L., Bao H., Yu C., Hu J., Ma F. (2022). Investigating *Phragmites australis* response to copper exposure using physiologic, Fourier Transform Infrared and metabolomic approaches. Funct. Plant Biol..

[81] Nolfi-Donegan D., Braganza A., Shiva S. (2020). Mitochondrial electron transport chain: Oxidative phosphorylation, oxidant production, and methods of measurement. Redox Biol..

[82] Yu H., Zhang L., Yang R., Jiang Y., Liao J., Chai S., Deng X., Wang L., Pu X., Zhang Y. (2023). Integrated Multiomics and Synergistic Functional Network Revealed the Mechanism in the Tolerance of Different Ecotypes of *Salvia miltiorrhiza* Bge. to Doxycycline Pollution. Environ. Sci. Technol..

[83] Zhang X., Gu C., Zhang T., Tong B., Zhang H., Wu Y., Yang C. (2020). Chloroplast (Cp) Transcriptome of *P. davidiana Dode* x *P. bolleana* Lauch provides insight into the Cp drought response and *Populus* Cp phylogeny. BMC Evol. Biol..

[84] Zhao Y., Yu H., Zhou J.M., Smith S.M., Li J. (2020). Malate Circulation: Linking Chloroplast Metabolism to Mitochondrial ROS. Trends Plant Sci..

[85] Zhou J.M., Zhang Y. (2020). Plant Immunity: Danger Perception and Signaling. Cell.

[86] He W., Chen C., Adedze Y.M.N., Dong X., Xi K., Sun Y., Dang T., Jin D. (2020). Multicentric origin and diversification of atp6-orf79-like structures reveal mitochondrial gene flows in *Oryza rufipogon* and *Oryza sativa*. Evol. Appl..

[87] Liu Y., Cox C.J., Wang W., Goffinet B. (2014). Mitochondrial phylogenomics of early land plants: Mitigating the effects of saturation, compositional heterogeneity, and codon-usage bias. Syst. Biol..

[88] Cui J.P., Wang R., Gu R.Q., Chen M.H., Wang Z.Y., Li L., Hong J.M., Cui S.X. (2024). Telomere-to-telomere *Phragmites australis* reference genome assembly with a B chromosome provides new insights into its evolution and polysaccharide biosynthesis. Res. Sq..

[89] Oh D.H., Kowalski K.P., Quach Q.N., Wijesinghege C., Tanford P., Dassanayake M., Clay K. (2022). Novel genome characteristics contribute to the invasiveness of *Phragmites australis* (common reed). Mol. Ecol..

[90] Lynch M., Koskella B., Schaack S. (2006). Mutation pressure and the evolution of organelle genomic architecture. Science.

[91] Chen Y.X., Chen Y.S., Shi C.M., Huang Z.B., Zhang Y., Li S.K., Li Y., Ye J., Yu C., Li Z. (2017). SOAPnuke: A MapReduce acceleration-supported software for integrated quality control and preprocessing of high-throughput sequencing data. Gigascience.

[92] Bi C., Shen F., Han F., Qu Y., Hou J., Xu K., Xu L.A., He W., Wu Z., Yin T. (2024). PMAT: An efficient plant mitogenome assembly toolkit using low-coverage HiFi sequencing data. Hortic. Res..

[93] Wick R.R., Schultz M.B., Zobel J., Holt K.E. (2015). Bandage: Interactive visualization of de novo genome assemblies. Bioinformatics.

[94] Altschul S.F., Gish W., Miller W., Myers E.W., Lipman D.J. (1990). Basic local alignment search tool. J. Mol. Biol..

[95] Li H. (2021). New strategies to improve minimap2 alignment accuracy. Bioinformatics.

[96] Shen W., Le S., Li Y., Hu F. (2016). SeqKit: A Cross-Platform and Ultrafast Toolkit for FASTA/Q File Manipulation. PLoS ONE.

[97] Li H. (2013). Aligning sequence reads, clone sequences and assembly contigs with BWA-MEM. arXiv.

[98] Danecek P., Bonfield J.K., Liddle J., Marshall J., Ohan V., Pollard M.O., Whitwham A., Keane T., McCarthy S.A., Davies R.M. (2021). Twelve years of SAMtools and BCFtools. Gigascience.

[99] Wick R.R., Judd L.M., Gorrie C.L., Holt K.E. (2017). Unicycler: Resolving bacterial genome assemblies from short and long sequencing reads. PLoS Comput. Biol..

[100] Chan P.P., Lin B.Y., Mak A.J., Lowe T.M. (2021). tRNAscan-SE 2.0: Improved detection and functional classification of transfer RNA genes. Nucleic Acids Res..

[101] Shi L.C., Chen H.M., Jiang M., Wang L.Q., Wu X., Huang L.F., Liu C. (2019). CPGAVAS2, an integrated plastome sequence annotator and analyzer. Nucleic Acids Res..

[102] Katoh K., Standley D.M. (2013). MAFFT Multiple Sequence Alignment Software Version 7: Improvements in Performance and Usability. Mol. Biol. Evol..

[103] Nguyen L.T., Schmidt H.A., von Haeseler A., Minh B.Q. (2015). IQ-TREE: A Fast and Effective Stochastic Algorithm for Estimating Maximum-Likelihood Phylogenies. Mol. Biol. Evol..

[104] Beier S., Thiel T., Münch T., Scholz U., Mascher M. (2017). MISA-web: A web server for microsatellite prediction. Bioinformatics.

[105] Benson G. (1999). Tandem repeats finder: A program to analyze DNA sequences. Nucleic Acids Res..

[106] Krzywinski M., Schein J., Birol I., Connors J., Gascoyne R., Horsman D., Jones S.J., Marra M.A. (2009). Circos: An information aesthetic for comparative genomics. Genome Res..

[107] Wang D.P., Zhang Y., Zhang Z., Zhu J., Yu J. (2010). KaKs_Calculator 2.0: A toolkit incorporating gamma-series methods and sliding window strategies. Genom. Proteom. Bioinform..

[108] Xiang C.Y., Gao F.L., Jakovlic I., Lei H.P., Hu Y., Zhang H., Zou H., Wang G.T., Zhang D. (2023). Using PhyloSuite for molecular phylogeny and tree-based analyses. Imeta.

[109] Chen C.J., Wu Y., Li J.W., Wang X., Zeng Z.H., Xu J., Liu Y.L., Feng J.T., Chen H., He Y.H. (2023). TBtools-II: A “one for all, all for one” bioinformatics platform for biological big-data mining. Mol. Plant.

[110] Edera A.A., Small I., Milone D.H., Sanchez-Puerta M.V. (2021). Deepred-Mt: Deep representation learning for predicting C-to-U RNA editing in plant mitochondria. Comput. Biol. Med..

[111] Picardi E., Pesole G. (2013). REDItools: High-throughput RNA editing detection made easy. Bioinformatics.

[112] Langmead B., Salzberg S.L. (2012). Fast gapped-read alignment with Bowtie 2. Nat. Methods.

[113] Love M.I., Huber W., Anders S. (2014). Moderated estimation of fold change and dispersion for RNA-seq data with DESeq2. Genome Biol..

[114] Sébastien Lê J.J., François H. (2008). FactoMineR: An R Package for Multivariate Analysis. J. Stat. Softw..

[115] Wickham H. (2016). ggplot2: Elegant Graphics for Data Analysis.

[116] Capella-Gutiérrez S., Silla-Martínez J.M., Gabaldón T. (2009). trimAl: A tool for automated alignment trimming in large-scale phylogenetic analyses. Bioinformatics.

[117] Kalyaanamoorthy S., Minh B.Q., Wong T.K.F., von Haeseler A., Jermiin L.S. (2017). ModelFinder: Fast model selection for accurate phylogenetic estimates. Nat. Methods.

[118] Ronquist F., Teslenko M., van der Mark P., Ayres D.L., Darling A., Höhna S., Larget B., Liu L., Suchard M.A., Huelsenbeck J.P. (2012). MrBayes 3.2: Efficient Bayesian phylogenetic inference and model choice across a large model space. Syst. Biol..

