# Supplementary figures and images for: Mitochondrial Genome Insights into Evolution and Gene Regulation in Phragmites australis
Source: Int J Mol Sci. 2025 Jan 10;26(2):546. doi: 10.3390/ijms26020546 (PMC11764873; doi:10.3390/ijms26020546)

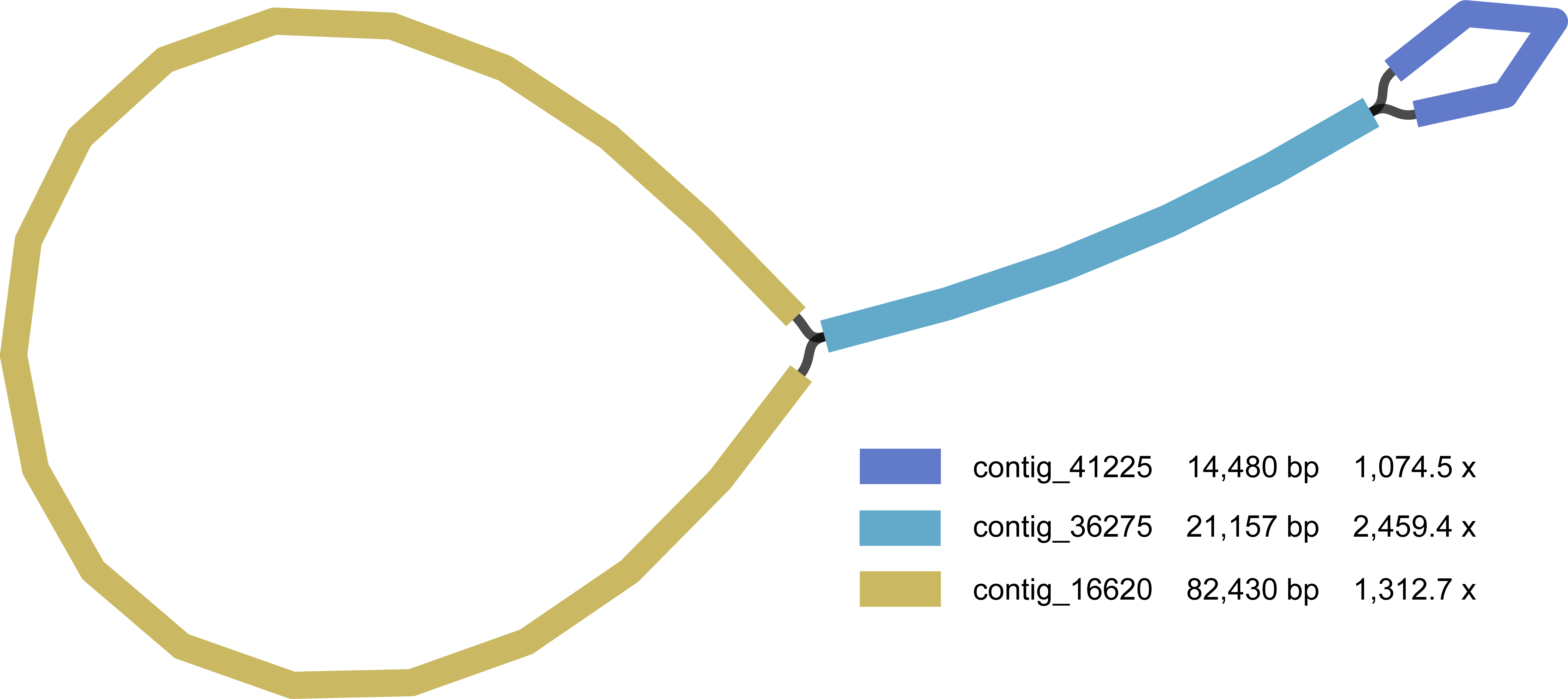

Supplement: Supplementary file 1 [file ijms-26-00546-s001.zip › supplementary files/Fig S1.tif]

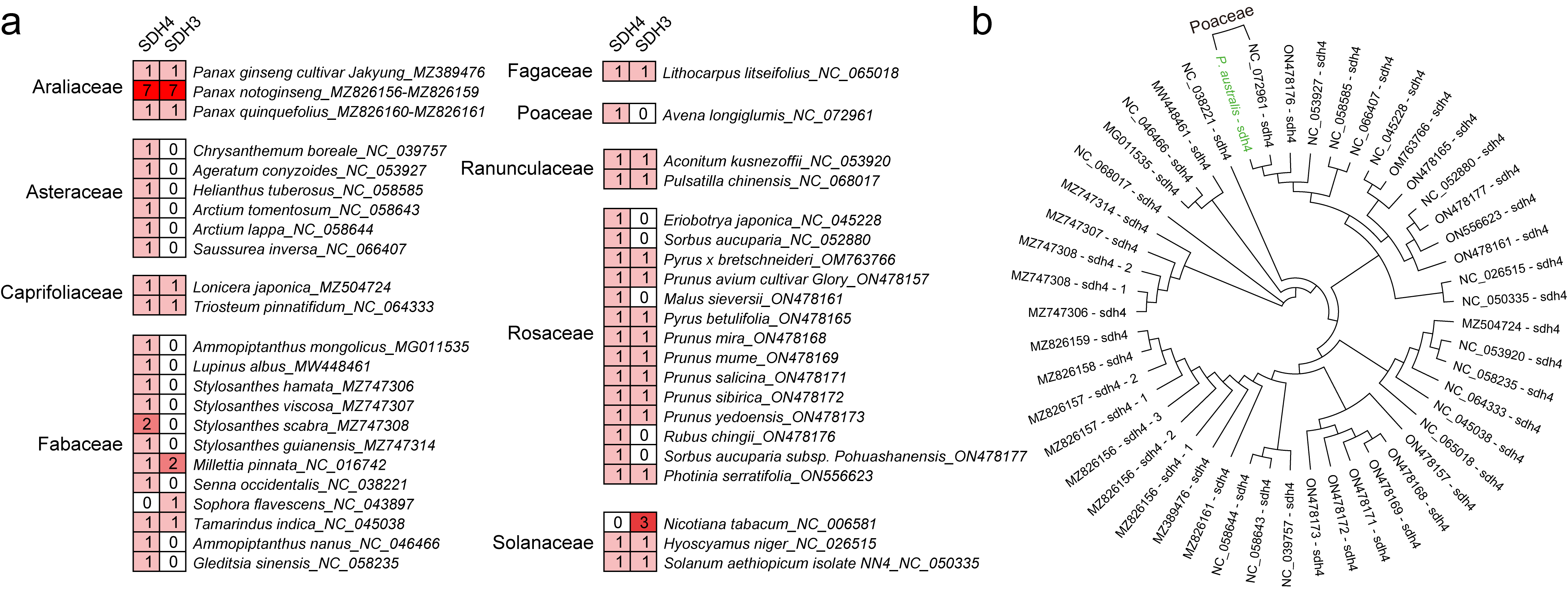

Supplement: Supplementary file 1 [file ijms-26-00546-s001.zip › supplementary files/Fig S3.tif]

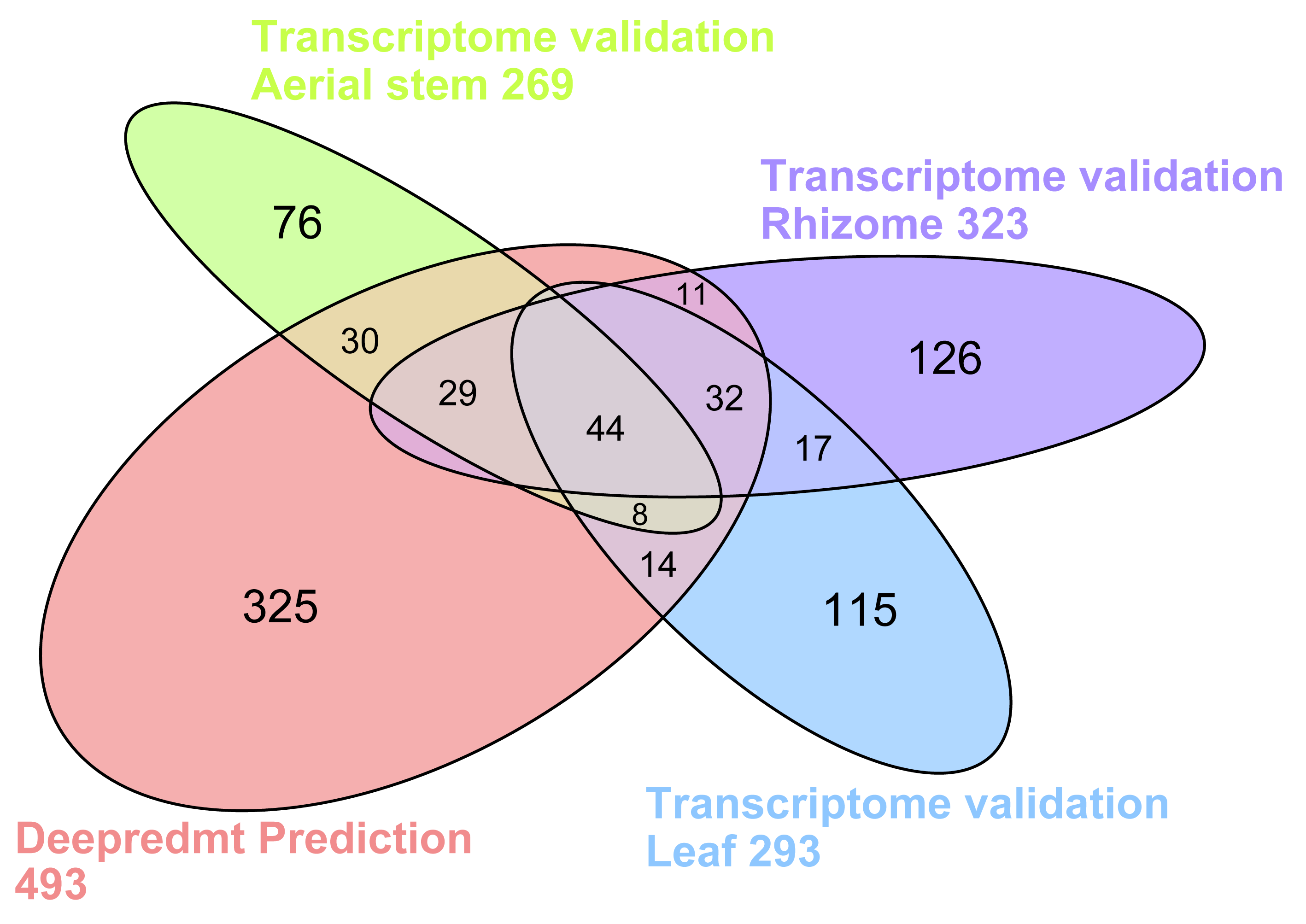

Supplement: Supplementary file 1 [file ijms-26-00546-s001.zip › supplementary files/Fig S5.tif]

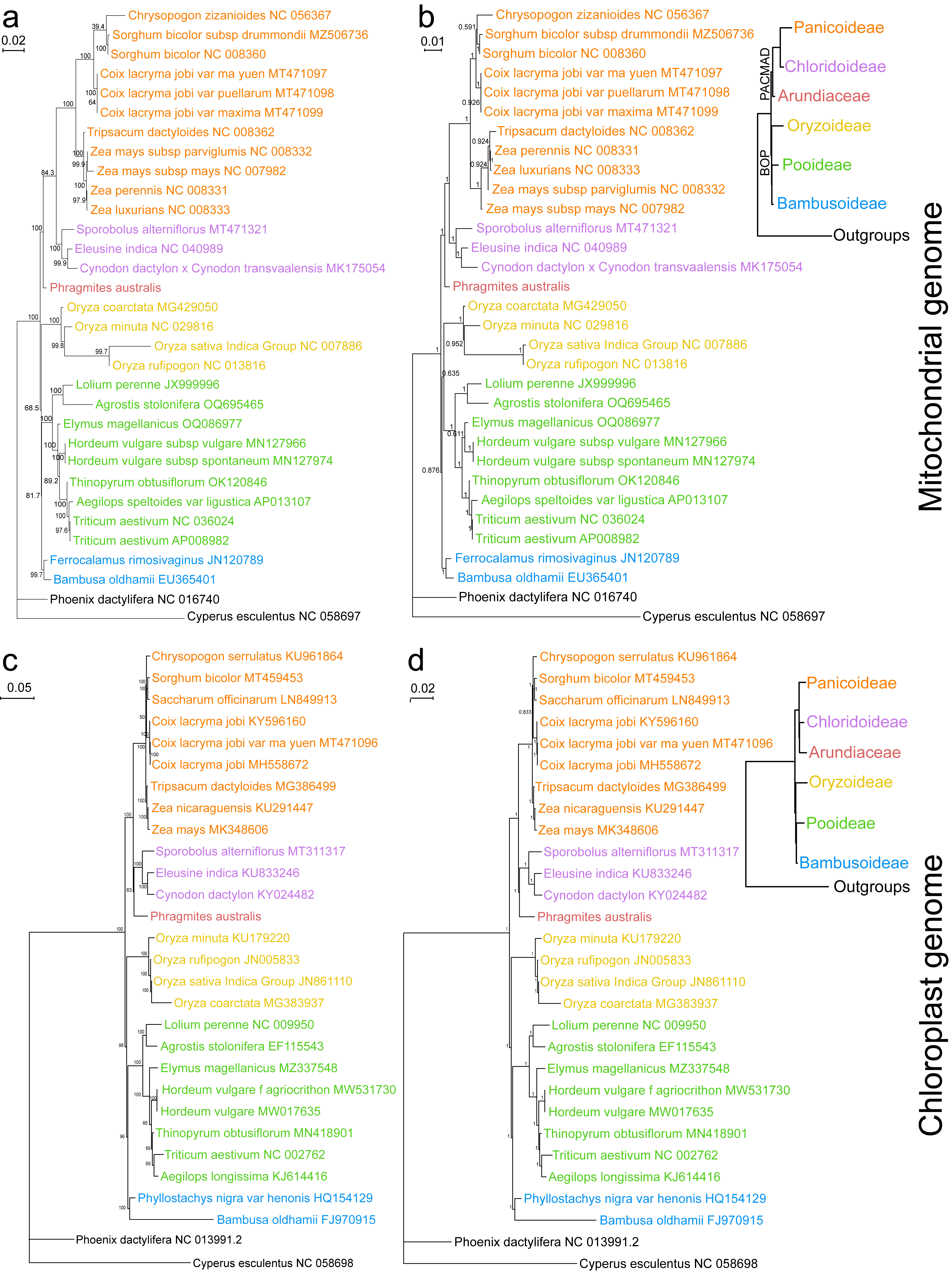

Supplement: Supplementary file 1 [file ijms-26-00546-s001.zip › supplementary files/Fig S6.tif]
